# JunB defines functional and structural integrity of the epidermo-pilosebaceous unit in the skin

**DOI:** 10.1038/s41467-018-05726-z

**Published:** 2018-08-24

**Authors:** Karmveer Singh, Emanuela Camera, Linda Krug, Abhijit Basu, Rajeev Kumar Pandey, Saira Munir, Meinhard Wlaschek, Stefan Kochanek, Marina Schorpp-Kistner, Mauro Picardo, Peter Angel, Catherin Niemann, Pallab Maity, Karin Scharffetter-Kochanek

**Affiliations:** 1 Department of Dermatology and Allergic Diseases, Ulm University, Ulm, 89081 Germany; 2 Aging Research Center (ARC), Ulm, 89081 Germany; 3 Laboratory of Cutaneous Physiopathology and Integrated Center of Metabolomics, San Gallicano Dermatologic Institute (IRCCS), Rome, 00144 Italy; 4 Department of Gene Therapy, Ulm University, Ulm, 89081 Germany; 5 Division of Signal Transduction and Growth Control, German Cancer Research Center (DKFZ) and DKFZ-ZMBH Alliance, 69120 Heidelberg, Germany; 6 Institute for Biochemistry II, University of Cologne, Cologne, 50931 Germany; 7 Center for Molecular Medicine Cologne, University of Cologne, Cologne, 50931 Germany

## Abstract

Transcription factors ensure skin homeostasis via tight regulation of distinct resident stem cells. Here we report that JunB, a member of the AP-1 transcription factor family, regulates epidermal stem cells and sebaceous glands through balancing proliferation and differentiation of progenitors and by suppressing lineage infidelity. JunB deficiency in basal progenitors results in a dermatitis-like syndrome resembling seborrheic dermatitis harboring structurally and functionally impaired sebaceous glands with a globally altered lipid profile. A fate switch occurs in a subset of JunB deficient epidermal progenitors during wound healing resulting in de novo formation of sebaceous glands. Dysregulated Notch signaling is identified to be causal for this phenotype. In fact, pharmacological inhibition of Notch signaling can efficiently restore the lineage drift, impaired epidermal differentiation and disrupted barrier function in JunB conditional knockout mice. These findings define an unprecedented role for JunB in epidermal-pilosebaceous stem cell homeostasis and its pathology.

## Abstract (web summary)

Epidermal homeostasis is maintained by the activity of stem cells. Here, the authors show that deficiency of the transcription factor JunB leads to altered Notch signaling in stem cells, resulting in a cell fate switch and de novo formation of aberrant sebaceous glands, altered epidermal differentiation and impaired barrier function.

## Introduction

Tissue homeostasis requires balanced cell proliferation and differentiation. This mainly depends on the fine-tuned regulation of resident stem cells (SC) which are endowed with the capacity to self-renew and—if required—differentiate into distinct lineages essential for the maintenance of tissue homeostasis^1^.

Mammalian epidermis offers a unique model tissue to investigate this highly regulated dynamic process of stem cell proliferation and differentiation, which comprises the interfollicular epidermis (IFE), hair follicles (HF), and the adjacent sebaceous glands (SG)^2–5^. The complex reciprocal interactions of transcription factors belonging to distinct cellular signaling pathways such as Wnt/β-catenin, Sonic Hedgehog, AP-1, TGFβ, and Notch in resident stem cells and their committed progenitors ensure proper developmental morphogenesis and postnatal maintenance of skin and its appendages^6–11^. Deregulation of transcription factors in mammalian skin has even been associated with the development and progression of various types of tumors^12,13^.

In recent years, members of the activator protein-1 (AP-1) transcription factor family have been linked to several skin pathologies^14,15^. AP-1, a dimeric transcription factor consisting of variable combinations of members of the Jun (c-Jun, JunB, JunD), Fos (Fos, FosB, Fra-1, and Fra-2), ATF, and Maf protein families, plays an important role in proliferation, differentiation and death^16,17^. In addition, JunB proteins are important regulators of cytokine expression and the immune response in skin^18,19^. Skin specific JunB deficiency in mice has been associated with numerous skin pathologies including psoriasis-like disease^18^, systemic lupus erythematosus, ulcerative skin lesions^20^, prolonged inflammation with delayed tissue remodeling^19^ and myeloproliferative disease^15^. These reports underscore the important role of JunB in skin homeostasis and protection from skin pathologies.

It is currently unresolved how JunB regulates fate identity, lineage plasticity, and behavior of epidermal stem cells residing in skin and its appendages during development and skin regeneration. To further dissect the role of JunB in lineage plasticity and fate identity, we have specifically deleted JunB in the basal epidermal progenitor cells of mouse skin, thus, targeting all putative stem cell populations within the IFE and its appendages.

We here uncover JunB as a multifaceted gate-keeper for skin homeostasis. JunB enforces its function by the previously unreported suppression of sebocyte fate decision from epidermal progenitors that—most likely via JunB-dependent regulation of Notch genes—are restricted to epidermal differentiation and, in consequence, maintain the protective barrier of the epidermis.

## Results

### JunB expression in mammalian skin under different conditions

AP-1 family members have been implicated in skin homeostasis especially in the regulation of terminally differentiated epidermal cells^21^. However, the contribution of distinct AP-1 family members, in particular JunB, during normal physiology and restitution of homeostasis following noxious insults has not been fully investigated.

**Fig. 1:**
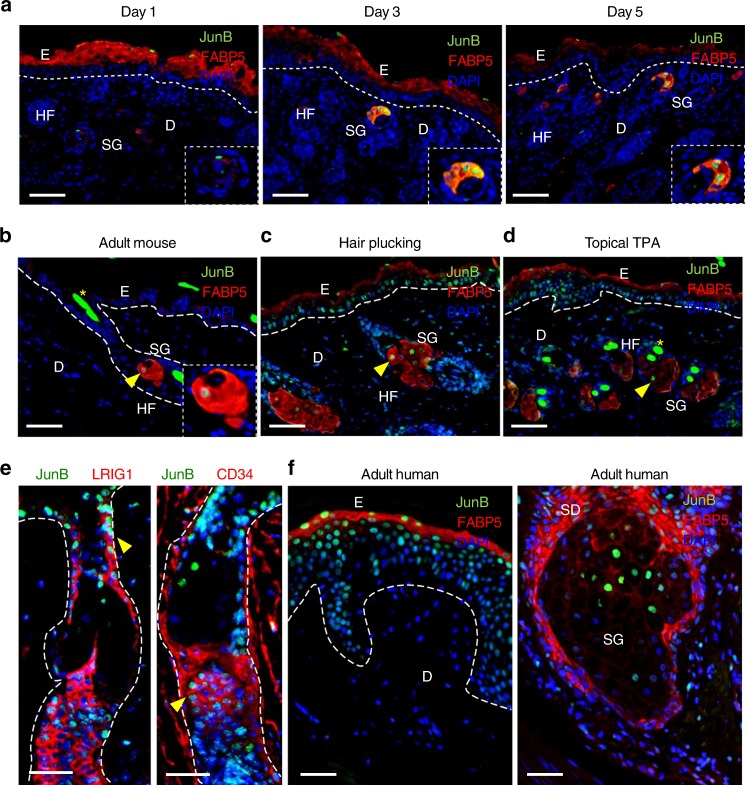
JunB expression during skin maturation and upon stress. **a** Representative microphotographs with double immunostaining for JunB (green) and FABP5 (red), indicative of sebaceous glands, in skin derived from 1, 3, and 5 days old mice. Inset showing magnified view of a sebaceous gland. Scale bars, 50 µm. **b** Immunostaining of JunB (green) and FABP5 (red) in 60 days old mice. Inset showing magnified view. **c** Representative microphotographs with double immunostaining of JunB (green) and FABP5 (red) in dorsal skin of 60 days old mice following hair plucking or **d** after topical TPA application, a potent inducer of proliferation. **e** Immunostaining of JunB (green) and LRIG1 or CD34 (red) in dorsal skin of 60 days old mice following hair plucking. **f** Immunostaining of JunB (green) and FABP5 (red) in adult human skin. E, epidermis; D, dermis; HF, hair follicle; SG, sebaceous gland; SD, sebaceous duct; HS, hair shaft. Asterisk indicates hair shaft autofluorescence. Dashed line indicates the epidermal-dermal junction. Scale bars, 50 µm

To assess the expression of JunB during maturation, we performed immunostaining of JunB in murine and human skin biopsies (Fig. 1a–f). JunB was strongly expressed in the upper skin layer largely harboring terminally differentiated epidermal cells (Fig. 1a–f). This finding is consistent with earlier reports^22,23^. Unexpectedly, we discovered an intense JunB staining in sebaceous gland at different time points during postnatal murine skin maturation (Fig. 1a, b).

In murine skin homeostasis a low rate of epidermal cell turnover was reported^24^. We here set out to analyze JunB expression under the condition of skin injury and hyperproliferation in which skin undergoes rapid cell proliferation and differentiation. Of note, the number of JunB positive cells substantially increased when skin was perturbed either by micro-injury through hair plucking or in response to topical TPA (12-O-Tetradecanoylphorbol 13-acetate) that triggers proliferation through activation PKC signaling^25^ (Fig. 1c, d). These potent stimuli significantly increased the expression of JunB not only in the sebaceous glands, transit amplifying cells (TAC) and committed progenitors of suprabasal layer, but also in undifferentiated basal epidermal progenitor cells and more defined hair follicle stem cells (HFSCs) including LRIG1 or CD34 positive populations (Fig. 1c–e, Supplementary Figure 1A and 1B). These findings were further confirmed at mRNA level employing qPCR analyses. In fact, we found an increased expression of specific JunB mRNA both in FACS sorted CD34^+ve^ and LRIG1^+ve^ stem cells in response to hair depilation (Supplementary Figure 1C and 1D). JunB expression was also detected in sebaceous glands and other epidermal cells of human adult skin under physiological condition (Fig. 1f). Our results strongly suggest a broader role of JunB in skin homeostasis and, previously unreported, JunB expression is not exclusively confined to the terminally differentiating epidermis.

To further dissect the role of JunB in the basal epidermal progenitor layer and the therein residing epidermal stem cells, we have generated basal epidermal progenitor cells specific homozygous JunB deficient mice using Cre recombinase driven by K14 promoter (hereafter JunB conditional knockout referred to as JunB cKO) (Supplementary Figure 2A, 2B and 2C). Surprisingly, JunB cKO mice were smaller in size and have reduced body weight compared to wild type mice (Supplementary Figure 2D and 2E). More importantly, adult (9–12 weeks) JunB cKO mice suffered from symptoms like itching and skin inflammation on facial and neck skin (Supplementary Figure 2D and 2F). As opposed to wild type mice, the fur of adult JunB cKO mice was damp most likely due to an excess glandular secretion or water loss from the skin (Supplementary Figure 2F). The severity of these symptoms gradually increased with age. Interestingly, this murine model with JunB deficiency in K14 expressing basal epidermal progenitors closely mirrors human seborrheic dermatitis clinically and histologically. Like human seborrheic dermatitis, our model revealed irregular alopecia (hair loss) presents with erythematous, and slightly moist skin covered by flaky scales (Supplementary Figure 2D and 2F). Likewise human seborrheic dermatitis, the JunB cKOs displayed typical histological features including sebaceous gland hyperplasia, epidermal hyperplasia with signs of spongiosis (edema between epidermal cells), parakeratosis and a persistence of infiltrating inflammatory cells (Supplementary Figure 2G, 3A, 3B and 3C). JunB cKO skin displayed an increased number of macrophages in the dermis and interfollicular epidermis, most likely reflecting the epidermal barrier defects (Supplementary Figure 3A). In addition, we assessed transepidermal water loss (TEWL) indicative of epidermal barrier function in JunB cKO skin using the TEWA meter. TEWL is inversely related to skin barrier function. Interestingly, we found enhanced epidermal water loss indicating poor barrier function in JunB cKO skin as opposed to wild type skin (Supplementary Figure 3D). Taken together, these findings indicate a critical role for JunB in skin homeostasis and—in case of JunB deletion—results in development of an inflammatory skin disorder sharing features with seborrheic dermatitis.

### Disturbed skin homeostasis in JunB cKO mice

**Fig. 2:**
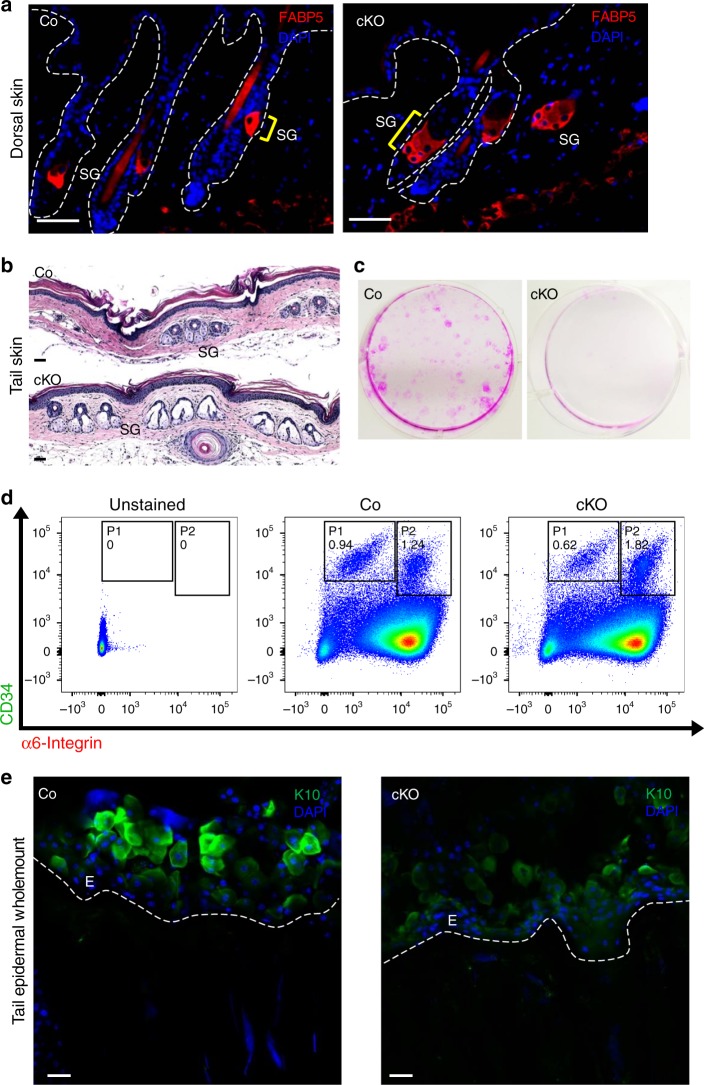
JunB cKOs display impaired skin homeostasis. **a** Representative photomicrographs with immunostaining of FABP5 (red), indicative of sebaceous glands, in dorsal back skin from wild type and JunB cKO mice. Nuclei stained with DAPI in blue. Scale bars, 50 µm. **b** Histology of tail skin from wild type and JunB cKO mice. Scale bars, 50 µm. **c** Representative photo of colony-forming unit assay from FACS purified HFSCs (CD34^+ve^α6Itg^Hi^) from wild type and JunB cKO mice back skin that were cultured in vitro for 2 weeks and then stained with Rhodamine B and Nile blue. *n* = 3 mice per genotype. **d** FACS analyses from second telogen phase displaying an increased ratio of P2 to P1 hair follicle stem cells (CD34^+ve^α6Itg^Hi^ /CD34^+ve^α6Itg^Low^) in unperturbed dorsal skin from 60 days old JunB cKO compared to wild type mice. **e** Confocal images of whole mount tail epidermis depict differentiated keratinocytes immunostained for the terminal differentiation marker k10 (green) in JunB cKO compared to wild type skin. Nuclei stained with DAPI in blue. Scale bars, 50 µm. E, epidermis; SG, sebaceous gland

Expanded sebaceous glands observed in the neck skin of JunB cKO mice prompted us to analyse their expression pattern and function in more detail. For this purpose skin and tail biopsies from non lesional skin of JunB cKO mice and wild type mice were subjected to H&E staining or immunostaining for the fatty acid binding protein 5 (FABP5), a key protein in sebaceous glands (Figs. 2a, b, Supplementary Figure 4A and 4B). Interestingly, sebaceous glands in JunB cKO mice were significantly enlarged as demonstrated by both H & E and FABP5 staining when compared to wild type mice (Figs. 2a, b, Supplementary Figure 4A and 4B). Next, we analyzed the activity and number of HFSCs. Interestingly, HFSCs (CD34^+ve^ alpha6-integrin^Hi^) isolated from JunB cKO mice skin formed significantly less colonies as opposed to wild type HFSCs (Fig. 2c and Supplementary Figure 4C), suggesting severely impaired differentiation and self-renewal in JunB cKO HFSCs. Though we did not find any significant difference in HFSCs numbers between JunB cKO and wild type mice, the numbers of double positive CD34^+ve^ alpha6-integrin^Hi^ were slightly higher in the skin of JunB cKOs (Fig. 2d). Of note, the ratio of CD34^+ve^ alpha6-integrin^Hi^ to CD34^+ve^ alpha6-integrin^Low^ HFSCs were increased in JunB cKO when compared to wild type mice (Fig. 2d, and Supplementary Figure 4D) indicating a defect in maintaining stem cell homeostasis within distinct subpopulations of bulge stem cells^26^. Together, these results underscore an essential role for JunB in the differentiation and maintenance of HFSCs under normal tissue homeostasis.

In the case of terminally differentiating epidermal cells a significantly reduced expression of keratin 10 or K10, involucrin and loricrin (markers for terminally differentiated keratinocytes) was found in JunB cKO mice tail and back skin when compared to wild type mice (Fig. 2e, Supplementary Figure 5A, 5B and 5C). These data are consistent with an earlier report suggesting an involvement of JunB in terminal differentiation^21^.

Thus, our results provide evidence for a crucial role of JunB in the regulation of sebaceous glands and epidermal compartment. In addition, our data strongly indicate an active role for JunB in skin homeostasis and—if dysregulated—may lead to impairment of hair follicle stem cells.

### Impaired healing and sebaceous gland hyperplasia in JunB cKOs

**Fig. 3:**
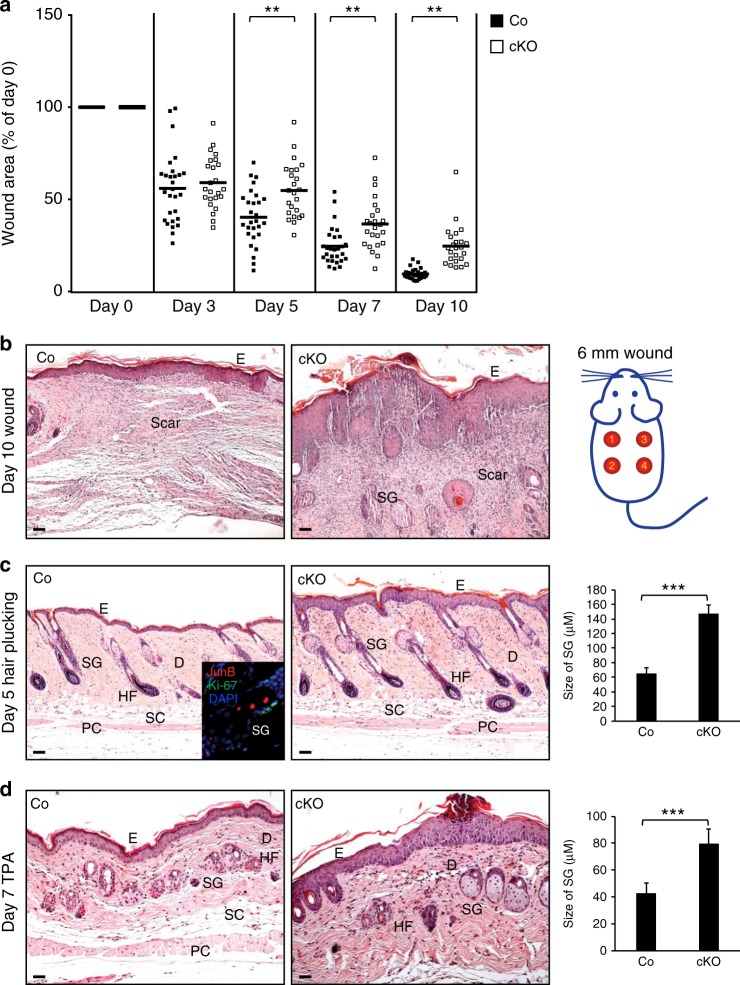
Impaired wound healing and stress response in JunB cKO mice. **a** JunB cKO mice display delayed wound healing compared to wild type mice following 6 mm full-thickness circular biopsy punch excision. **P < 0.001, one-way ANOVA (*n* = 7) **b** Representative H&E photomicrographs of a 10 days wound depicting a hyperplastic and thickened epidermis and enlarged sebaceous glands in JunB cKO mice compared to wild type mice. Scale bars, 50 µm. **c** Enhanced epidermal and sebaceous gland hyperplasia in JunB cKOs at day 5 following hair plucking. Inset image depicts opposing role of JunB (red) on proliferating cells marked in green with Ki67. Quantification of the size of sebaceous glands following hair plucking, ***P < 0.001, *t*-test (*n* = 3). **d** The topical application of TPA, a strong enhancer of proliferation, for 7 days markedly triggered epidermal and sebaceous gland hyperplasia. Quantification of the size of sebaceous glands after TPA application, ***P < 0.001, t-test (*n* = 3). E, epidermis; D, dermis; HF, hair follicle; SG, sebaceous gland; SC, subcutaneous layer; PC, panniculus carnosus. Scale bars, 50 µm

To assess whether JunB deficiency interferes with the regenerative capacity of the skin, we induced macro- and micro-injuries in wild type and JunB cKO mice. Interestingly, delayed healing of full thickness wounds was observed in JunB cKO mice at day 5, 7, and 10 post-wounding as compared to wild type mice (Fig. 3a, b). A hyperplastic thickened epidermis with a parakeratotic stratum corneum and enhanced inflammation was observed in JunB cKOs as compared to wild type and uninjured JunB cKO skin (Fig. 3b and Supplementary Figure 4A). In addition, we detected impressively enlarged sebaceous glands at wound sites in JunB cKOs (Fig. 3b). These results indicate an essential so far unreported role of JunB in the regulation of keratinocytes and their progenitors during skin repair and regeneration.

Similarly, micro-injuries due to hair plucking significantly induced hyperplasia of the epidermis and sebaceous glands in JunB cKO (Fig. 3c). Sebaceous glands of JunB cKOs adopted a balloon-like morphology, while they remain restricted to relatively smaller in size in wild type mice (Fig. 3c). These results imply that JunB maintains the structural integrity of the skin most likely by concomitant fine-tuning of proliferation and differentiation.

JunB has been described as a tumor suppressing protein in earlier reports^14,27^. Therefore, we assessed whether JunB may also suppress epidermal hyperproliferation essential for tumor initiation. Notably, TPA, a tumor-initiating agent, strongly induced hyperplasia not only in the epidermis but also in sebaceous glands of JunB cKO as compared to wild type mice (Fig. 3d), thus confirming its anti-proliferative, possibly tumor suppressing effects. These results are in line with our earlier findings showing an upregulation of JunB following TPA challenge (Fig. 1d).

Notably, we observed a significantly higher proliferation in keratinocyte progenitors residing in the basal epidermal layer and in LRIG1 positive junctional zone stem cells indicative of sebaceous gland precursors in JunB cKOs as opposed to significantly less proliferation of these epidermal cells in the skin of wild type mice (Supplementary Figure 6A and 6B). As micro-injury employed by hair plucking stimulated JunB expression in skin (Fig. 1c), we wondered whether JunB expression inversely correlated with cell proliferation. Co-staining for JunB and the proliferation marker Ki67 after hair plucking in wild type mice skin showed that most of the proliferating Ki67 positive cells, confined to the basal layer or the hair follicle growth zone of wild type mice skin, were distinctly negative for JunB (Supplementary Figure 6C and inset Fig. 3c). By contrast, JunB expression was observed in Ki67 negative progeny cells close to hair follicle growth zone or in the suprabasal layer of epidermis from wild type mice skin (Supplementary Figure 6C and inset Fig. 3c). These results provide evidence for a suppressive role of JunB on proliferation of skin progenitor cells.

In summary, our results underscore the JunB role in the reinstitution of skin homeostasis during wound repair and tumor progression through tight regulation of epidermal progenitors and their differentiated progeny.

### De novo sebaceous gland formation in JunB cKO wound epidermis

**Fig. 4:**
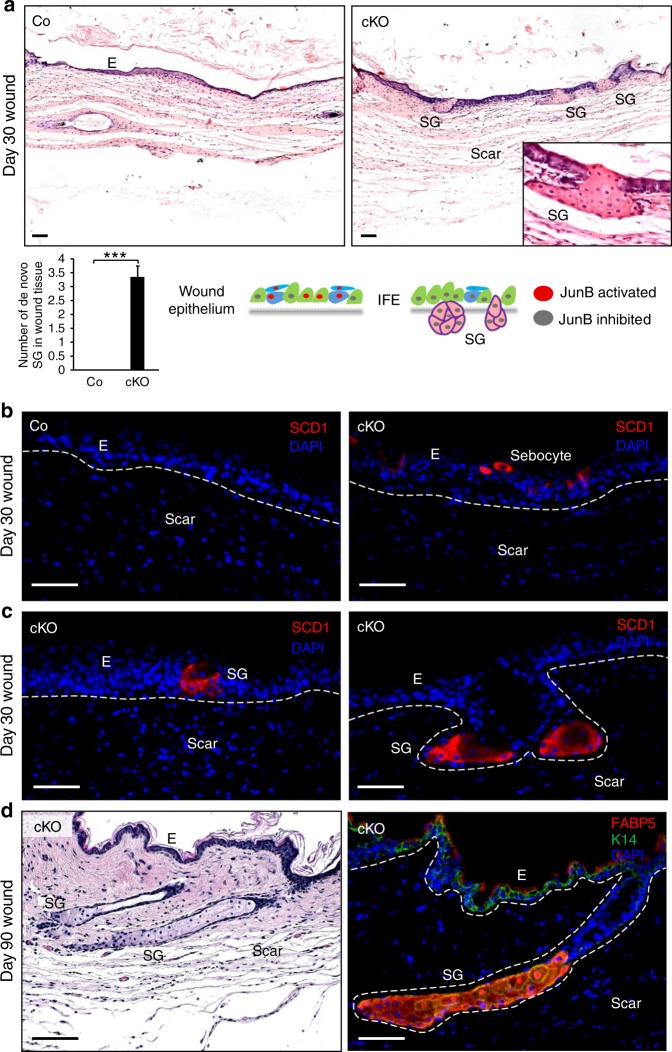
De novo formation of sebaceous glands in JunB cKO wounds. **a** Representative H&E photomicrographs of 30 days old wound restoration tissue depicting ectopic sebaceous glands in JunB cKO mice. Inset showing magnified view. E, epidermis; SG, sebaceous gland. Scale bars, 50 µm. Quantification of de novo sebaceous gland numbers in 30 days old wounds. ***P < 0.001, *t*-test (*n* = 3). Graphical sketch summarizes JunB-dependent suppression of epidermal progenitor differentiation towards sebocyte lineage (left bottom panel as opposed to lineage plasticity of epidermal progenitors towards sebocyte differentiation (right bottom panel). **b**, **c** Representative microphotographs with immunostaining of SCD1 (red), indicative of sebocyte differentiation, demonstrating different stages of de novo formation of sebaceous glands in 30 days old wounds in JunB cKO mice. Nuclei stained with DAPI in blue. **d** Representative photomicrographs displaying the formation of elongated sebaceous duct in 90-day-old wound epithelium in JunB cKO mice. Wound tissues were stained either with H & E or the sebaceous glands specific marker FABP5 (red) and the basal layer specific keratin K14 (green). Nuclei stained with DAPI in blue. Scale bars, 50 µm. E, epidermis; SG, sebaceous gland

To determine the fate and behavior of JunB cKO basal keratinocyte and keratinocyte progenitors during skin regeneration, we followed wound healing for an extended period of 30 days. Surprisingly, we observed the formation of ectopic sebaceous glands originating from the regenerating wound epidermis in JunB cKO, indicating a switch in the differentiation program in a subset of epidermal progenitors cells lacking JunB thus favoring sebaceous gland specification (Fig. 4a). These results were further supported by immunostaining of Stearoyl-CoA desaturase-1 (SCD1), a sebaceous gland specific marker, in restored wound tissue of JunB cKOs 30 days after wounding. Sebocytes were detected as single cells (Fig. 4b), as conglomerate of several sebocytes within the epidermis (Fig. 4c) or as entire sebaceous gland (Fig. 4c) reflecting different stages of sebaceous gland development in JunB cKO mice. Even though transient lineage infidelity has recently been described in regenerating epidermis during wound repair^11,28^, where hair follicle or sebaceous gland duct stem cells leave the niche and migrate towards the wound bed, a plasticity switch towards sebocyte has so far not been reported. Furthermore, to determine whether these de novo sebaceous glands may in long term generate hair follicles or sebaceous gland tumors, we monitored JunB cKO wounds for 90 days. We observed that the de novo sebaceous glands further elongated into a tube shaped duct devoid of any other skin appendages (Fig. 4d). Sox9 expression—an established marker for hair follicle stem cells—was detected in newly formed sebaceous glands and their adjacent cells as opposed to wild type mice wounds (Supplementary Figure 7A). This finding hints to the origin of de novo sebaceous glands. However, further lineage tracing experiments targeting distinct skin stem cells in conjunction with JunB deficiency are needed to support this conclusion in future experiments. Intriguingly, a high expression of JunB was observed virtually in every keratinocyte present in regenerating wound epidermis of wild type mice compared to unwounded skin (Supplementary Figure 7B). The higher expression of JunB most likely restricts the plasticity of keratinocyte progenitor’s subpopulation towards sebaceous glands during tissue repair (Supplementary Figure 7B). Together, these results imply JunB to play an important role in fate determination and regulation of stem cell plasticity of keratinocyte precursors and—if dysregulated—leads to lineage drift, impaired skin regeneration and wound healing.

### JunB deficiency altered the lipid profile of cKO hairs

**Fig. 5:**
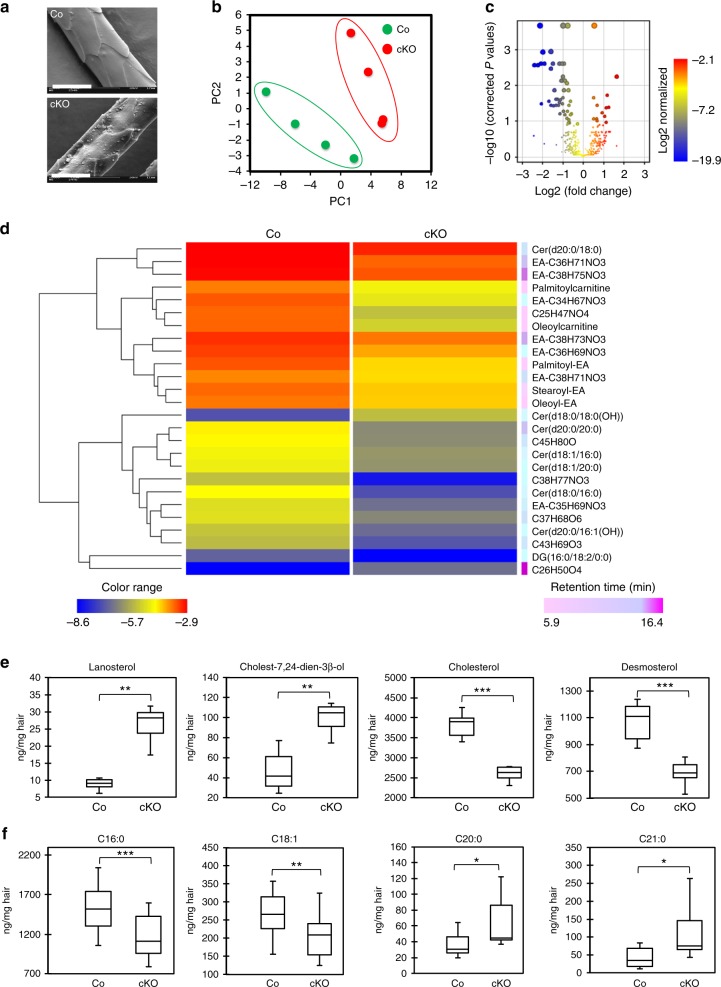
Substantially altered sebum components in JunB cKO hairs. **a** Scanning electron microscopic images demonstrate irregular hair architecture with a rough carved surface in JunB cKO mice, magnification 2000×. Scale bars, 50 µm. **b** Principal component analysis (PCA) illustrating the variances between the different sample groups. **c** Volcano plot displaying significantly changed lipid species between wild type and JunB cKO mice hair extracts. Color scale reflects Log 2 normalized values. **d** Integrated heatmap analyses of significantly changed lipid species between wild type and JunB cKO mice hair extracts. Color scale (blue to red) reflects Log 2 values and color scale (pink to purple) shows retention time in the LC-MS analysis indicated in minutes. **e** GC-MS analyses of hair extracts from adult wild type and JunB cKO mice depicting lipid species involved in cholesterol biosynthesis or **f** different classes of free fatty acid. *P < 0.05, **P < 0.01, ***P < 0.001 (*n* = 3), t-test. Upper border of box depict 3rd quartile, while lower border depict 1st quartile. The midline represents the median value and the whiskers represents the maximum and minimum values

As sebaceous glands constitute an integral component of the hair unit ensuring the generation of fully functional hairs, we examined whether hair quality is influenced by the abnormal sebaceous glands of JunB cKO mice. Scanning electron microscopy of hairs from JunB cKO demonstrated an abnormal architecture with a rough and scaly surface compared to the smooth and glossy hair of wild type mice (Fig. 5a), suggesting detrimental consequences of impaired sebaceous glands on hair quality. Next, we set out to explore the possibility whether biosynthesis of sebum lipids providing protective hydrophobic barrier on hairs and outer skin surface is impaired. Indeed, GC-MS in conjunction with untargeted HPLC-MS of hair extracts revealed a global shift in lipid composition of JunB cKO compared to wild type mice (Fig. 5b–f).

Untargeted HPLC-MS demonstrated substantial differences in unbiased comprehensive lipid profiles between wild type and JunB cKO hair extracts (Fig. 5b–d). Volcano plot analysis, in fact, revealed 46 differently bio-synthesized lipid entities between wild type and JunB cKO hair extracts, among them 26 with a more than two fold change (Fig. 5c). Heatmap analysis of lipid species modified with a fold change ≥ 2 identified a substantial decrease in the abundance of acylcarnitine (palmitoyl- and oleoylcarnitine), endocannabinoids (palmitoyl-, oleoyl-, and stearoylethanolamide) in JunB cKO hair extracts compared to wild type (Fig. 5d). To the best of our knowledge neither the secretion of acylcarnitine and endocanabinoids nor their JunB-dependent decrease in mice sebum have previously been reported. The identity of acylcarnitine and endocannabinoids, bearing carnitine and ethanolamine (EA) head groups, respectively, was confirmed by comparison of MS/MS spectra with authentic compounds and in silico spectra available on the human metabolome database (http://www.hmdb.ca/). Furthermore, we analyzed the LC-MS data for the EA-bearing lipid compounds, which reports retention time (RT), annotations, elemental formula, ionization, and results of the MS/MS experiment for the detected EA family members (Supplementary Table 1). The fragmentation of the 538, 552, and 566 parent ions generated the fragments 300, 282, and 62, which overlapped the features of palmitoyl-EA MS/MS spectrum (Supplementary Table 1). Similarly, fragments common to the oleoyl-EA were generated from the 590 and 592 parent ions, whereas fragments of stearoyl-EA were generated from the 564, 566, 592, and 594 parent ions (Supplementary Table 1). Thus, the spectra of the 566 and 592 ions revealed that each one appeared to be present in two major isoforms. As represented in Fig. 5d, levels of acylcarnitine, endocannabinoids, EA-related compounds, and ceramides were significantly decreased in JunB cKO mice.

In addition, as opposed to wild type hair extracts, we observed a significant decrease in the concentrations of cholesterol and desmosterol with a significant increase of upstream metabolites such as cholest-7,24-dien-3β-ol and lanosterol in JunB cKO (Fig. 5e). These data provide evidence for a severe impairment of cholesterol biosynthesis most likely due to the disruption of the responsible *Kandutsch-Russel and Bloch* pathways in JunB cKO sebaceous glands^29^. Moreover, biosynthesis of free fatty acids was also affected in JunB cKO hairs as GC-MS analyses of hair extracts revealed a significant decline in C16:0 and C18:1, while a significant increase in the concentration of free fatty acids C20:0 and C21:0 when compared to wild type hair extracts (Fig. 5f).

In summary, we observed a severely disrupted biosynthesis of sebum components reflecting functional impairment of sebaceous glands, which in conjunction with impaired differentiation and persistent inflammation contribute to skin barrier dysfunction and the observed hair pathology in JunB cKO mice.

### Globally altered gene expression profile in JunB cKO skin

**Fig. 6:**
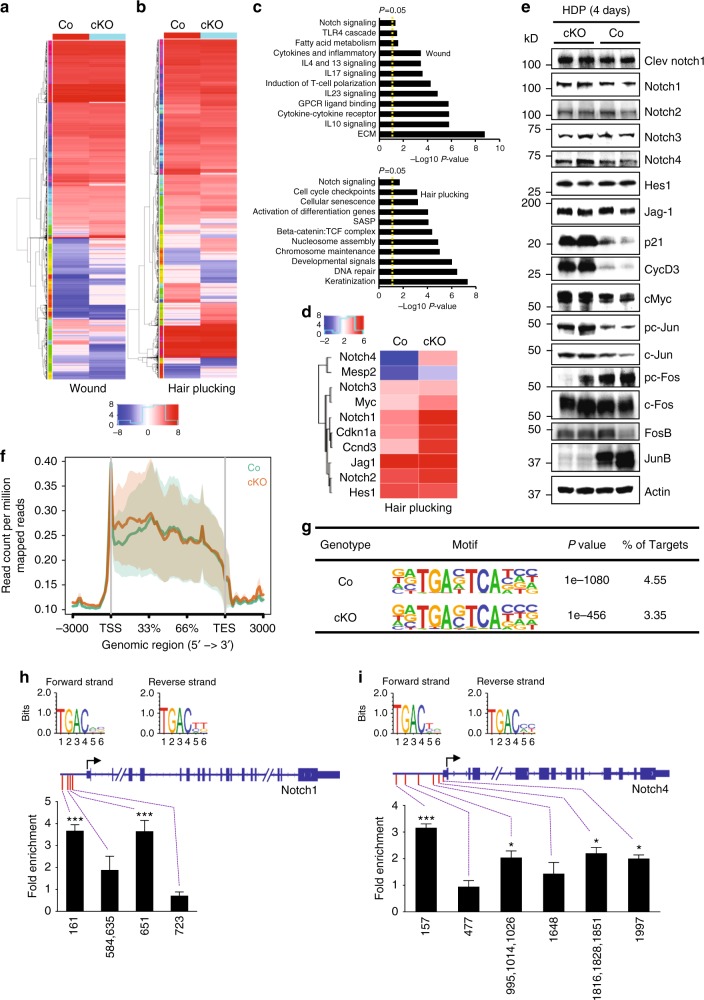
JunB deficiency significantly altered the global transcriptome. **a**, **b** Heatmap depicting transcriptome profiling of samples (*n* = 3) from wild type and JunB cKO skin harvested after wounding or hair plucking. The color reflects the log2 scale of relative expression as in the case of Figure (**a**, **b**, and **d**). **c** Pathway enrichment analyses depicting highly upregulated pathways in JunB cKO mice skin compared to wild type after wounding or hair plucking. Dashed line represents *P* value of 0.05. *P* < 0.05 for the pathway enrichment was considered significant. **d** Heatmap depicting genes involved in Notch signaling from wild type and JunB cKO skin collected post hair plucking. **e** Western blot analyses of key activated Notch pathway proteins and AP-1 members 4 days post hair plucking in JunB cKO and wild type skin. **f** Distribution of ATAC-seq signals across the gene body of the whole mouse genome and **g** high enrichment of the AP-1 motif both in primary epidermal progenitor cells isolated from JunB cKO and wild type skin post hair plucking. **h** ChIP assay from wild type epidermal progenitor cells isolated after hair depilation displays distinct JunB binding sites in *Notch1* and **i** *Notch4* promoter regions. The fold enrichment is calculated by comparing the Ct values of each primer sets (JunB/AP1 binding site) with a JunB/AP1 negative region (3′ UTR in case of *Notch1* and 5′ UTR in case of *Notch4*). Each of the Ct values of either JunB/AP1 binding sites or negative region was subtracted from respective IgG Ct values (*n* = 3), *P < 0.05, ***P < 0.001, *t*-test. The position of motif (JunB binding site) is mentioned in the X axis. A schematic representation of JunB/AP1 binding sites (Red bar) in the promoter region of either *Notch1* or *Notch4* gene is presented above the graphs

To comprehensively assess global gene expression changes under normal as well as stress conditions like wounding and hair plucking, dorsal skin from epidermal JunB cKO and wild type mice were subjected to transcriptome analyses. RNA-seq analyses revealed considerable differences in gene expression between JunB cKO and wild type uninjured skin or skin harvested after wounding and hair plucking, respectively (Fig. 6a, b, Supplementary Figure 8A and 8B). As expected, we observed a marked increase in pro-inflammatory genes from JunB cKO wounds as compared to wild type wounds. A significant upregulation in pathways involved in cytokine and chemokine signaling as well as extracellular matrix (ECM) remodeling was detected by pathway enrichment analyses in JunB cKO wounds (Fig. 6a, c). Hair depilation represents a rather pro-developmental condition with less inflammation. Among other pathways, upregulation of “Notch signaling” was particularly intriguing (Fig. 6c, d) as canonical Notch signaling is a major regulator of epidermal differentiation^6^, which may trigger aberrant differentiation in JunB cKO skin. We further confirmed Notch involvement at protein level. Western blot analyses showed substantial Notch activation and its target proteins such as p21, CyD3, and cMyc in JunB cKO skin in response to hair plucking as opposed to wild type skin (Fig. 6e, Supplementary Figure 9A and Supplementary Figure 12). In addition, RNA-seq analyses—under unperturbed conditions—also uncovered considerable differences in gene expression between JunB cKO and wild type skin (isolated from dorsal skin of 60-days-old mice) (Supplementary Figure 8A and 8B). Among others, we identified a significant decrease in genes involved in lipid metabolism of sebaceous glands (Supplementary Figure 8C) most likely affecting lipid synthesis and sebaceous gland maturation, a finding which is in line with our earlier lipidomic findings (Fig. 5b–f). In addition, immunostaining of Awat2—the key enzyme in wax synthesis localized in mature sebaceous glands—showed a significantly reduced expression in JunB cKO mice (Supplementary Figure 8G). By contrast, genes involved in mucin synthesis such as *MUC1*, *MUC 2*, *MUC 4*, and *MUC 6* were highly upregulated in JunB cKO mice (Supplementary Figure 8C). In case of *Wnt* family genes, which are major determinants of epidermal stemness in skin, a significantly increased expression of *Wnt5b*, *7a*, *8b*, *9a*, and *9b* in JunB cKO primary epidermal progenitor cells (Supplementary Figure 8D) was observed. JunB deficiency also modulated the expression of *Notch* genes (Supplementary Figure 8E) and several inflammatory mediators (Supplementary Figure 8F). Of note, we found a marked alteration in the expression of other AP-1 assembly transcription factors in JunB cKO skin (Fig. 6e and Supplementary Figure 9A). This implies that JunB plays a crucial role in the stability or most likely activity of other AP-1 complex members.

Next, to discover the potential role for JunB/AP1 in the regulation of differentially expressed genes and accessibility to gene-regulatory chromatin regions, an assay for transposase accessible chromatin with high-throughput sequencing (ATAC-seq) analysis was employed. ATAC-seq signals from epidermal progenitor cells isolated either from JunB cKOs or wild type mice after hair plucking were mapped over the whole genomic region to detect the impact of JunB deficiency on the chromatin state. A marked enrichment of ATAC-seq signals at transcript start sites (TSS) in JunB cKO epidermal progenitor cells were detected as opposed to wild type (Fig. 6f, Supplementary Figure 10A and 10B), which most likely influence global gene transcription. Interestingly, ATAC-seq analyses revealed that AP-1 motif was among the top enriched open chromatin domains both in JunB cKO and wild type mice skin following hair plucking (Fig. 6g). Furthermore, the ATAC-seq de novo motif analyses from JunB cKO mice has revealed marked enrichment of the AP-1 motif in both upregulated and downregulated genes (Supplementary Figure 9B), suggesting a key regulatory role for AP-1 in maintaining epidermal homeostasis. Consistent with our findings, ATAC-seq analyses of the previously deposited data, GEO: GSE89928^11^ uncovered a substantial enrichment of AP-1 during epidermal wound healing (24.55%) compared to homeostatic epidermal (15.31%) and HF stem cells (4.73%) (Supplementary Figure 9C).

As the Notch pathway is highly activated in JunB cKO mice, we further explored whether JunB interacts with Notch promoter regions. *In-silico* analysis, indeed, revealed potential JunB binding sites in the promoter region of several Notch family genes (Supplementary Figure 10C). ChIP assays were subsequently performed to determine direct physical interaction between JunB and the Notch promoter in wild type epidermal progenitor cells in response to hair plucking. Of note, multiple binding sites for JunB in the promoter region of *Notch 1* (Fig. 6h), and *Notch 4* gene (Fig. 6i) were confirmed, indicating direct regulation of Notch signaling by JunB in the epidermis. Of note, the ChIP-qPCR assay demonstrated a reduction in repressive histone marks (H3K27Me3 and H3K9Me3) and a gain of active histone marks (H3K9Ac and H3K4Me) within the promoter region of *Notch1* and *Notch4* gene in JunB cKO basal epidermal progenitor cells as compared to control (Supplementary Figure 11A and 11B).

In aggregate, our results clearly establish JunB to be essential for maintenance as well as fate restriction of distinct stem and progenitor cells residing in the skin.

### Notch inhibition restored skin homeostasis in JunB cKOs

**Fig. 7:**
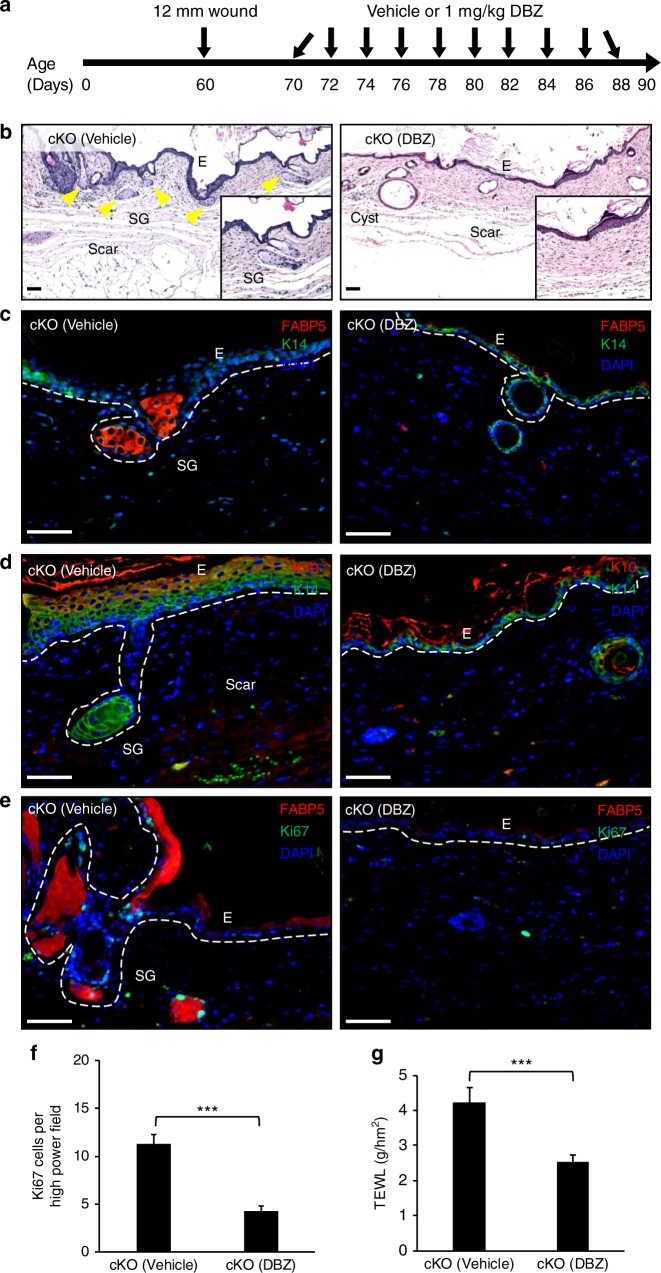
Notch inhibition restored epidermal homeostasis in JunB cKOs. **a** Cartoon depicting experimental design and administration schedule of the Notch inhibitor DBZ or vehicle in JunB cKO mice. **b** Representative H&E staining and **c** immunostaining for the sebaceous gland specific marker FABP5 demonstrates the absence of de novo sebaceous gland formation in newly formed wound epithelium of the Notch inhibitor DBZ treated JunB cKO mice as opposed to vehicle treated group (*n* = 3). Scale bars, 50 µm. **d** Representative microphotographs with immunostaining of differentiated keratinocytes marker K10 (red) and undifferentiated keratinocytes marker K14 (green), in DBZ treated JunB cKO mice compared to vehicle treated group (*n* = 3). Nuclei stained with DAPI in blue. Scale bars, 50 µm. **e** Representative microphotographs with immunostaining of proliferation marker Ki67 (green) and FABP5 (red) and **f** quantification, in DBZ treated JunB cKO mice as opposed to vehicle treated group. ***P < 0.001, *t*-test (*n* = 3). Nuclei stained with DAPI in blue. Scale bars, 50 µm. **g** TEWL, which is inversely related with epidermal barrier function, was measured on the dorsal skin of DBZ treated JunB cKO mice and vehicle treated group (*n* = 3). ***P < 0.001, *t*-test

To further explore whether upregulated Notch signaling in JunB cKO mice is causal for the observed lineage drift and impaired skin function, we used a pharmacological approach to inhibit Notch signaling in JunB cKO mice. We repetitively treated JunB cKO mice either with vehicle or with the gamma-secretase inhibitor dibenzazepine (DBZ, 1 mg/kg)^30^, a specific Notch inhibitor (Fig. 7a). Gamma-secretase facilitates the final cleavage step of the precursor form of Notch and thereby activates Notch signaling. Employing DBZ during wound healing, we examined lineage plasticity, differentiation, proliferation and barrier function in JunB cKO mice.

Interestingly, a complete absence of ectopic sebaceous glands was uncovered in regenerating wound epithelium in the DBZ treated group as compared to the vehicle treated JunB cKO mice (Fig. 7b, c). Instead, DBZ treated JunB cKOs showed thin scar epithelium and the presence of keratin cysts that were typically reported in Notch deficient mice^31^. These data confirm effective Notch blockade with DBZ in our model system (Fig. 7b, c). This was further supported at protein level (Supplementary Figure 11C). In addition, chronic DBZ administration restored the expression of the terminal differentiation marker K10 and confined the expression of undifferentiated keratinocytes marker K14 to the basal layer in JunB cKO skin as compared to vehicle treated group (Fig. 7d). Consistent with these results Notch inhibition reduced epithelial cell proliferation in JunB cKO skin, as shown by a marked reduction of the proliferation marker Ki67 (Fig. 7e, f). To study whether long term Notch blockade can rescue the barrier function in JunB cKO skin, TEWL measurements were performed on the dorsal skin of mice. In the DBZ treated mice a significant reduction in TEWL was found when compared to the vehicle treated group (Fig. 7g). These data indicate a marked restoration of the impaired barrier function in JunB cKO skin.

In aggregate, our results revealed beneficial action of Notch inhibition in JunB cKO skin that not only oppose the lineage drift, but also restored the epidermal homeostasis and skin function.

## Discussion

**Fig. 8:**
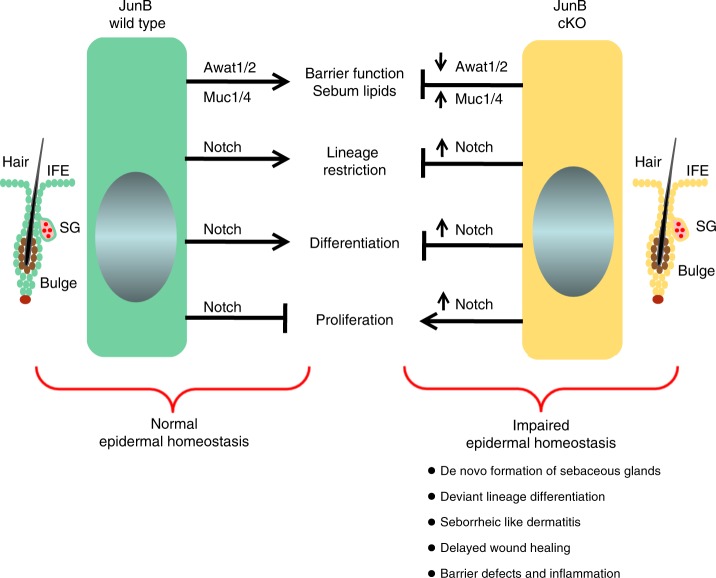
Graphical summary describing JunB function in skin. Schematic drawing depicts wild type epidermal cells and their progenitors expressing K14 (left panel, green) and the JunB cKO epidermal cells and their progenitors (right panel, light yellow) in their different epidermal skin compartments (IFE, interfollicular epidermis; SG, sebaceous gland). In the JunB wild type conditions—via Notch signaling—epidermal differentiation and proliferation of cells are balanced even upon different stimuli. This in conjunction with the restriction of epidermal progenitor toward epidermal differentiation guarantees a functional epidermal barrier. By contrast, in the JunB cKO situation, epidermal cells and their progenitors—due to overactivation of Notch signaling—cannot undergo proper differentiation but instead are caught in unrestrained proliferation. Most importantly, JunB cKO epidermal progenitors are not lineage restricted and tweak into the sebocyte differentiation and under conditions of skin irritation/trauma result in de novo sebaceous gland formation. The lineage deviation of epidermal progenitors towards as sebocyte phenotype is accompanied with a functional change and in consequence an increase in the synthesis of mucin at the expense of lipids. In fact, the major enzyme catalyzing wax ester synthesis is decreased. This together with the lack of epidermal differentiation and a change in the outermost composition of the wax impairs the protective barrier of the epidermis. Impairment of JunB function at several molecular and cellular levels results in complex phenotypes like seborrheic like dermatitis, delayed wound healing and loss of skin homeostasis

Our study highlights a novel role of JunB in skin that is responsible for fate/lineage plasticity of stem cells located in the interfollicular epidermis and upper pilosebaceous compartment. We uncovered JunB as a multifaceted gate-keeper of skin homeostasis (see graphical summary Fig. 8). JunB enforces its function by the previously unreported suppression of sebocyte fate decision from epidermal progenitors, which via JunB-dependent tight regulation of *Notch* genes are restricted to epidermal differentiation, and in consequence maintain the multilayered protective barrier of the epidermis. In addition, JunB was identified to serve the maintenance of the protective epidermal barrier by inducing lipid synthesis in sebaceous glands. Distinct classes of lipids produced from sebaceous glands seal the internal moisture of the skin and display microbicidal functions^32^.

The key role of epidermal JunB in skin homeostasis was supported by complex pathologies in the absence of JunB in cKO mice. First, a dermatitis-like syndrome closely mimicking human seborrheic dermatitis^33,34^ spontaneously occurred in mechanically stressed anatomical sites. JunB cKOs suffering from seborrheic dermatitis revealed enlarged sebaceous glands most likely due to the lack of fate restriction of epidermal progenitor cells towards sebocytes or to hyperproliferation. Second, partly due to enhanced *Notch* genes expression in JunB cKOs, differentiation of epidermal progenitors is impaired in favor of an unlimited proliferation resulting in a hyperplastic epidermis with a parakeratotic stratum corneum (persisting nuclei of epidermal cells in the stratum corneum). Third, the protective barrier of the skin is broken as deduced from the clinical picture of a moist skin surface covered by flaky scales. Fourth, perturbing skin by full-thickness (epidermis and dermis) wounds, hair plucking or topical TPA treatment resulted in an increase in the size of sebaceous glands. In fact, healing and regeneration of full thickness wounds were markedly delayed in JunB cKO mice. Our findings together with an earlier report that demonstrated wound healing defects in fibroblast-specific JunB deficient mice^19^, strongly implicate an essential role for JunB both in epidermis and dermis to restore skin homeostasis after injury. Our major, previously unreported finding was the de novo formation of sebocytes and sebaceous glands from renewing wound epidermis of JunB cKO mice, strongly indicating a deviation from the normal differentiation program in the subset of JunB deficient progenitors. These so called “free” (not attached to hair follicles) lipid producing glands are typically found in eyelids, oral epithelium, and ears^35^. Our data provide first evidence that JunB plays a major role in the restriction of epidermal stem cell fate decisions and suppression of inflammation during tissue repair and regeneration.

JunB cKOs display the typical symptoms of seborrheic dermatitis which are partly overlapping with earlier described Keratin 5 specific epidermal JunB single^15^ or JunB/cJun double deficient mice^18^, in terms of epidermal hyperplasia and skin inflammation. Transgenic mice published in the above reports suffer from severe arthritis (inflammation of the joints), strong skin inflammation, scaling and alopecia in irregular skin areas including ears and the tail. In contrast to these reports, our study specifically addresses the regulatory role of JunB in sebaceous gland homeostasis and in controlling lineage plasticity of epidermal stem cells in regenerating skin.

Using comprehensive transcriptome analysis, we uncovered potential JunB-dependent targets that—due to disrupted expression—most likely dysregulate tissue homeostasis in epidermal specific JunB cKO mice. Among them, activated Notch signaling in JunB cKOs was particularly interesting as balanced Notch signaling is essential for proper epidermal differentiation^6^. Our results identified a specific enrichment of the JunB/AP1 motif during epidermal differentiation, which directly suppresses Notch signaling via physical interaction with the *Notch1* and *Notch4* promoters. AP-1 has also been implicated in transcriptional repression of matrix metalloproteinase-9 through recruitment of histone deacetylase-1 in response to interferon β^36^ and 17a-hydroxylase-17,20-lyase via blocking transcriptional activity of the nuclear receptor steroidogenic factor-1^37^. In addition, anti-proliferative actions of JunB/AP1 family have been reported to be due to direct activation and induced expression of the cell cycle check point marker, p16^38^. Strikingly, blocking unrestrained Notch activation by pharmacologic means rescued epidermal homeostasis through suppressing lineage drift towards de novo sebaceous glands and epidermal proliferation, while improving differentiation and barrier function in JunB cKO mice.

In addition, we identified down regulation of genes involved in fat metabolism such as *Awat1*, *Awat2*, *Dgat1*, and *Scd3*. Altered lipid biosynthesis as uncovered by an unbiased lipidomic approach together with reduced free fatty acid and cholesterol/desmosterol production from JunB cKO sebocytes not only affect hair quality, but also disrupt the protective hydrophobic barrier layer of the epidermis and hair. Our findings highlight an important role for JunB both in the regulation of lineage plasticity, proliferation as well as in the control of sebaceous gland and epidermal barrier function.

In terms of disease pathologies, JunB cKO mice in conjunction with aberrant Notch signaling revealed several features overlapping with seborrheic dermatitis^33,34^. The present study thus may be highly relevant to pathological conditions related to malfunction of sebaceous gland like seborrheic dermatitis. Our findings hold substantial promise to develop new therapeutic targets for patients suffering from sebaceous gland associated pathologies.

## Methods

### Generation of basal epidermal cells specific JunB cKO mice

We have generated basal epidermal progenitor cell-specific JunB cKO mice by crossing mice carrying cre recombinase under the control of K14 promoter (B6; K14 Cre) with mice harboring *JunB* floxed alleles (B6; JunBtmAngl). *JunB* deletion was confirmed by standard PCR using specific primers (Supplementary Table 2). Mouse colonies were housed in a certified animal facility in accordance with European guidelines. The local ethical committee (Regierungspräsidium Tübingen) approved these experiments.

### FACS analysis and colony-forming unit assay of HFSCs

For FACS analysis, freshly isolated primary epidermal progenitor cells from adult skin were stained for 30 min on ice with APC-conjugated anti-α6-integrin (Biolegend, 313616, dilution 1:100) and FITC-conjugated anti-CD34 (eBioscience, 11-0341-85, dilution 1:50) antibodies. FACS-analysis was performed on a BD FACSCanto^TM^ II and data were analyzed with FlowJo software. While FACS sorting and colony-forming unit assay (CFU) assay was performed as previously described^39^. For CFU assay, FACS sorted HFSCs (CD34^+ve^α6Itg^Hi^) either from control or cKO mice (*n* = 3) were cultured in 6 well plates over the period of 14 days under standard in vitro conditions.

### Wound healing study

Prior to wounding, mice were anesthetized and four 6 mm full excisional thickness wounds were induced on shaved back skin using standard biopsy punches. Wound size was photographically documented on day 0, 3, 5, 7, and 10 and quantified using Adobe Photoshop Elements 9 software (Adobe Systems). At the end of study wound tissue was harvested and stored either in −80 °C or fixed in 4% paraformaldehyde.

### Hair plucking assay

Hairs from mice back skin were depilated on P54 (postnatal day 54) using a mechanical epilator. On P58 (postnatal day 58) or 4 days after hair depilation, skin tissue was harvested and fixed in 4% paraformaldehyde or snap frozen in liquid nitrogen.

### TPA-induced hyperproliferation

TPA (12-O-Tetradecanoylphorbol 13-acetate, Sigma-Aldrich) was dissolved in DMSO at 1 mg/ml and further diluted in sterile acetone to final concentration of 20 nM. Diluted TPA (20 nM) was then topically applied twice daily on an alternate day over a week on the shaved back skin. At day 7, TPA or mock-treated mice were sacrificed and skin were processed for histological analyses.

### Whole mount staining and confocal imaging of tail epidermis

Pieces of skin tail were incubated in trypsin at 37 °C for 2 h. The epidermis was separated from the dermis as an intact sheet and subsequently washed with PBS and fixed in 4% paraformaldehyde at 4 °C for 2 h. Before immunostaining, epidermis sheets were washed and incubated in blocking buffer (1% BSA, 5% goat serum, 0.8% Triton in PBS) for 4 h at room temperature on a rocking plate. Epidermal sheets were then incubated in primary antibodies FABP5 (R&D systems, AF1476, dilution 1:500) to mark sebaceous glands or K10 (Covance, PRB-159P, dilution 1:100) to detect differentiated keratinocytes overnight at 4 °C on the rocking plate. After washing with PBS for 2 h samples were then incubated with appropriate secondary antibodies overnight at 4 °C on the rocking plate. Tail epidermis sheets were then washed with PBS for 2 h. Nuclei were stained in DAPI for 15 min and mounted in DAKO fluorescent mounting medium. Images were captured using Zeiss confocal microscope^40^. Approximately 10–15 optical sections per sample were done and subsequently processed with ImageJ.

### Western blot analyses and immunostaining

Western blot analyses and immunostaining were performed as previously described^41^. Western blotting was performed with following primary antibodies (1:1000): Anti- Clev Notch 1, Anti-Notch 1, Anti-Hes-1, Anti-P21, Anti-cMYc, and Anti-Cyclin D3 (Cell Signaling Technology, Notch activated targets antibody sampler kit, 68309), Anti-Notch4 (Millipore, 07-189), Anti-pc-Jun (Cell Signaling Technology, 3270), Anti-cJun (Cell Signaling Technology, 9165), Anti-JunB (Cell Signaling Technology, 3753) and secondary antibodies (goat anti-rabbit, 111-035-045 or goat anti-mouse 115-035-003, Jackson Immuno Research Inc., dilution 1:10,000). For immunostaining, tissue sections were incubated with following primary antibodies: Anti-JunB (Cell Signaling Technology, 3753, dilution 1:100), Anti-FABP5 (R&D systems, AF1476, dilution 1:1000), Anti-SCD1 (Cell Signaling Technology, 2438), Anti-CD34 (eBioscience, 11-0341-85, dilution 1:200), Anti-LRIG1 (R&D systems, AF3688, dilution 1:25), Anti-K14 (Covance, PRB-155P, dilution 1:50,000), Anti-K10 (Covance, PRB-159P, dilution 1:100), Anti-Ki67 (Thermo Fisher Scientific, MA5-14520, 1:50), Anti-F4/80 (eBioscience, 14-4801-81, dilution 1:200), Anti-Ly6C (BD Pharmingen, 550291, dilution 1:200), Anti-CD18 (eBioscience, 14-0181-81, dilution 1:200), Anti-Involucrin (Covance, PRB-140C, dilution 1:200), Anti-Loricrin (Biolegend, 905104, dilution 1:200), Anti-Sox9 (Millipore, AB5535, dilution 1:200) and Awat2 (Abcam, ab204904, dilution 1:200). Alexa Fluor 488 conjugated goat anti-rabbit IgG (Invitrogen, A11008), Alexa Fluor 555 conjugated goat anti-rabbit IgG (Invitrogen, A21428), Alexa Fluor 488 conjugated goat anti-mouse IgG (Invitrogen, A11001), Alexa Fluor 555 conjugated donkey anti-goat IgG (Invitrogen, A21432) and Alexa Fluor 488 conjugated goat anti-Rat IgG (Invitrogen, A11006) (dilution 1:1000) were used as secondary antibodies.

### Scanning electron microscopy

Hairs were plucked and mounted on aluminum pin stub covered with conductive carbon adhesive tabs. Sputter coating was performed with gold. Hairs were then analyzed under a field emission scanning electron microscope (ZEISS DSM 962) at 10 kV.

### Comprehensive transcriptome profiling and quantitative PCR

Total RNA from all the experimental groups was isolated using RNeasy mini kit (Qiagen). Total RNA from three different samples of the same group (either injured or intact skin from wild type and JunB cKO mice) were pooled and 5 µg of this pooled total RNA was used to deplete rRNA using a commercially available kit (Ribo-Zero gold, Illumina) following the manufacturer’s instructions. The rRNA depleted samples were subjected to library preparation using TruSeq Stranded Total RNA Library Prep Kit (Illumina). The library was sequenced on an Illumina NextSeq 500 (Microsynth AG, Switzerland) using NextSeq v2 run for RNA sequencing (1 × 75 bp reads). The demultiplex data were adapter trimmed and quality filtered using FASTX-toolkit (http://hannonlab.cshl.edu/fastx_toolkit/index.html). Adapter trimmed and quality filtered data was next employed to analyze the differential expression of genes using Tuxedo pipeline^42^. In brief, the unpaired quality filter and adapter trimmer reads were first mapped with tophat (version 2.1.0)/HISAT2 to the mouse genome (GRCm38) using Bowtie2/ HISAT2 index, respectively. Mapped data were then used for transcript assembly using Cufflinks (version 2.2.1) and finally differentially expressed genes were analyzed using Cuffdiff. All commands for FASTX-toolkit, Tophat and cufflinks were entered into a script file to automate the whole process of analysis. The script can be obtained from the authors on request. The heatmap of the expression data was generated using gplots in RStudio environment. The gene ontology and pathway analyses were performed using IPA (Qiagen).

### Notch inhibition study

For Notch inhibition experiments, 10 days after inflicting two 12 mm wounds on the back of mice, 1 mg/kg DBZ or dibenzazepine ((S)-2-(2-(3,5-difluorophenyl)acetamido)-N-((S)-5-methyl-6-oxo-6,7-dihydro-5H-dibenzo[b,d]azepin-7-yl)propanamide, SYNCOM, Netherlands) or vehicle (0.1% Tween-80, 0.5% methylcellulose) was orally administered to JunB cKO mice on alternate days for 20 days. Mice were then analysed for skin barrier function followed by harvesting wounds and skin for subsequent analyses.

### Quantitative GS-MS and untargeted LC-MS analysis of hair lipids

Equally weighed hair specimens from wild type and JunB cKO mice were extracted with chloroform/methanol mixture 2:1 after addition of the 10 nmole hexadeuterated cholesterol (d6-cholesterol) (CDN isotopes, Canada) and 1 nmole N-palmitoyl-d31-d-erythro-sphingosine (d31-Cer(d18:1/16:0) (Avanti Polar Lipids, USA). as the internal standards (IS). Aliquots of redissolved lipid extracts were analyzed by GC-MS for the quantification of free fatty acids and cholesterol/congeners following derivatization with 100 µl BSTFA-1% trimethylchlorosilane (TMCS) in pyridine. The redissolved lipid extracts were further analyzed by untargeted HPLC-ESI-MS (time of flight, TOF) in positive ion mode.

The separation in GC-MS was performed with the 30 m–0.250 mm (i.d.) GC DB-5MS UI fused silica column (Agilent Technologies) chemically bound with a 5% diphenyl 95% dimethylpolysiloxane cross-linked stationary phase (0.25 mm film thickness). Helium was used as the carrier gas. Samples were acquired in scan mode by means of electron impact (EI) MS (Agilent Technologies). Quantitation of lipid metabolites detected with the GC-MS method was performed against calibrations curves of authentic free fatty acids and sterols using d6-cholesterol as the internal standard. Quantitative results were normalized by the mg of extracted hair and reported as ng/mg hair.

The separation in LC-MS was operated with the chromatographic apparatus consisting of a 1200 series rapid resolution HPLC (Agilent Technologies) equipped with a degasser, autosampler, and thermostated column compartment from the same manufacturer. For the reversed phase HPLC separation a Zorbax SB-C8 rapid resolution cartridge 2.1 × 30 mm 3.5 µm p.s. (Agilent Technologies) was used. Hair extracts were eluted with a binary gradient of (A): 5 mM ammonium formate in MilliQ water (18.2 Ω) and (B): methanol/2-propanol 95/5. The mobile phases were filtered through 0.45 μm glass filters and continuously degassed under vacuum. The elution program was as follows: 0–2 min 60 % B, 18 min 99% B, 18–24 min 99% B, 30 min 60% B. The flow rate was maintained at 0.6 mL/min during the entire HPLC run. The column was thermostated at 60 °C. The injection volume was 1 μl. The eluent outlet was connected to two different MS analyzers for detection and characterization.

Measurements of accurate mass and isotope pattern were conducted with a G6220A series TOF-MS (Agilent Technologies, Germany) equipped with an ESI interface. Nitrogen was used as the nebulizing and desolvation gas. The temperature and the flow of the drying gas were 350 °C and 10 l/min, respectively. The capillary and the cone voltage were 4000 and 80 V, respectively. Scan mode TOF mass spectra were acquired in the positive ion mode by using the TOF at 10,000 mass resolving power for scans over the *m/z* range from 100 to 1200. MS scans were processed using the Mass Hunter software (B.01.03 version). To enhance accurate mass measurement for the ion species, a reference solution was vaporized in continuum in the spray chamber. The resulting data were converted to mass centroid from which the accurate *m/z* value was measured.

The results of the untargeted approach were normalized by the internal standards and the weight of each sample. In particular, following acquisition, lists of detected compounds associated with HPLC-MS data (retention time, accurate mass, and peak area) were generated by each data file with the Mass Profinder software (Agilent Technologies). The compounds lists were then converted into a compound exchange files and treated with Mass Profiler Professional software (Agilent Technologies). Internal and external normalization were done against peak area of internal standards and hair weight in each extract. Differences between co and JunB cKO mice were searched in hair specimens with univariate methods (volcano plot). Hierarchical clustering of samples was searched on the basis of the relevant discriminating compounds. Identification of compounds was performed on the basis of the accurate mass detected with the TOF MS and the MS/MS spectra generated with a triple quadrupole MS in separate run. ESI tandem mass spectra were obtained with a G6410A series triple quadrupole (Agilent Technologies). Data were acquired in the positive ion mode at unit mass resolving power. MS spectra were averaged and processed with the Mass Hunter software (B.01.03 version). The collision energy (CE) applied was 26 V, while the fragmentor (F) voltage was set at 140 V.

### Transposase-accessible chromatin sequencing analysis

The transposase-accessible chromatin sequencing (ATAC-seq) assay was performed on epidermal progenitor cells harvested 4 days after hair plucking from wild type and JunB cKO mice skin as previously described^43^. In brief, 100,000 epidermal progenitor cells were lysed in ATAC lysis buffer (10 mM Tris-HCl, pH 7.4, 10 mM NaCl, 3 mM MgCl2, 0.1% NP-40) for 1 min followed by nuclei isolation using centrifugation at 500 × *g* for 10 min at 4 °C. The isolated nuclei were then subjected to transposition reaction with Tn5 transposase (Illumina) for 30 min at 37 °C. DNA was then isolated using Qiagen MinElute kit and subjected to PCR reaction to add the barcode using Nextera index kit (Illumina). The PCR products were then purified by 1.8x AMPure XP bead and analyzed for quality. Finally, the libraries were sequenced on Illumina NextSeq 500 using NextSeq v2 chemistry (2 × 75 cycles).

ATAC-seq analysis was performed on our results and a recently published data^11^ using the workflows described earlier^43,44^ with slight modifications. In brief, the paired end data were aligned to mouse genome (mm10) using Bowtie2 (version 2.3.0)^45^. The aligned sam files were converted to quality filtered bam file using samtools (version 1.3.1). The duplicated reads were then removed using Picards tools MarkDuplicates command (version 2.9.2). The reads of mitochondrial DNA from the quality filtered deduplicated bam files were removed using samtools idxstats command followed by conversion to bed file. As suggested earlier, the Tn5 transposase binds as a dimer and inserts two adapters separated by 9 bp, all the reads those aligned to +ve strand were offset by +4 bp, and all those reads aligned to the –ve strand were offset −5 bp using a customized bash script. The shifted bed files were next used to find the peaks using callpeak command of MACS2 (version 2.1.1). The peaks were finally annotated with annotatePeaks command and motif search were performed by findMotifsGenome command of Homer (version 4.9).

### ChIP assay

Chromatin immunoprecipitations (ChIP) were performed in epidermal progenitor cells after hair depilation using commercially available kits (truChIP Chromatin Shearing Kit Covaris and SimpleChIP kit, Cell Signaling). Briefly, 10 × 10^6^ epidermal progenitor cells were subjected to fixation, nuclei preparation and chromatin fragmentation using truChIP Chromatin Shearing Kit (Covaris). Cells were fixed with 1% formaldehyde for 10 min and chromatin shearing was done using high cell chromatin shearing protocol (PIP 75, Duty factor (%) 10, CPB 200, time 7 min) of M220 Focused-ultrasonicator (Covaris) in milliTUBE–1 ml. Sheared chromatin was then centrifuged for 15 min at 18,000 × *g* and 4 °C. Next, 10 µg of sheared chromatin from each samples were used for ChIP. In each samples excluding IgG control, 10 µl of ChIP grade JunB antibody or specific histone antibody (H3K27Me3, H3K9Me3, H3K9Ac and H3K4Me) (Cell signaling technologies) was added. In the IgG control, 10 µl of rabbit IgG was added. Thereafter both samples were incubated at 4 °C overnight with continuous rotation. Next, 30 µl of ChIP-grade protein G magnetic beads were added in each sample followed by 2 h incubation with continuous rotation at 4 °C. The beads were then washed with low and high salt buffer and chromatin was eluted from the beads with 150 µl ChIP Elution Buffer after incubating for 30 min at 65 °C. The eluted chromatin was reversed cross-links and DNA were isolated by Qiagen MinElute kit. Binding of JunB to *Notch1* and *Notch4* promoters was quantified by qPCR, where 2 µl of ChIP DNA was used per qPCR reaction. Employed ChIP primers for the detection of JunB binding to *Notch-1* and *Notch-4* promoter regions are listed in Supplementary Table 3.

### Assessment of transepidermal water loss

Transepidermal water loss (TEWL) was measured with a Tewameter TM 300 display device (Courage and Khazaka, Cologne, Germany) on the shaved mice skin.

### In silico analysis of putative JunB binding sites on the promoter regions

Searching of putative JunB/AP1 binding sites in the promoter regions of Notch genes was performed by PROMO, which uses TRANSFAC version 8.3. The sequence logo was built using weblogo command of RWeblogo package.

### Statistical calculations

Error bars represent SEM. The significance of differences between two groups was analyzed by Student’s *t*-test or one-way ANOVA, followed by Bonferroni correction for comparing the difference between more than two groups and presented as *P < 0.05, **P < 0.01, or ***P < 0.001.

## Electronic supplementary material

Supplementary Information

Peer Revies File

## Data Availability

The RNA-Seq and ATAC-Seq data used in this present study were deposited in the Gene Expression Omnibus (GEO) with accession number ‘GSE113688’ and in Sequence read archive (SRA) with accession number ‘SRP142509’, respectively.
