# Supplementary material for: JunB defines functional and structural integrity of the epidermo-pilosebaceous unit in the skin
Source: Nat Commun. 2018 Aug 24;9:3425. doi: 10.1038/s41467-018-05726-z (PMC6109099; doi:10.1038/s41467-018-05726-z)
Supplement: Supplementary file 1 — Supplementary Information [file 41467_2018_5726_MOESM1_ESM.pdf]

Supplementary Information

JunB defines functional and structural integrity of the epidermo-pilosebaceous unit in the skin

Singh et al.

Supplementary Figure 1

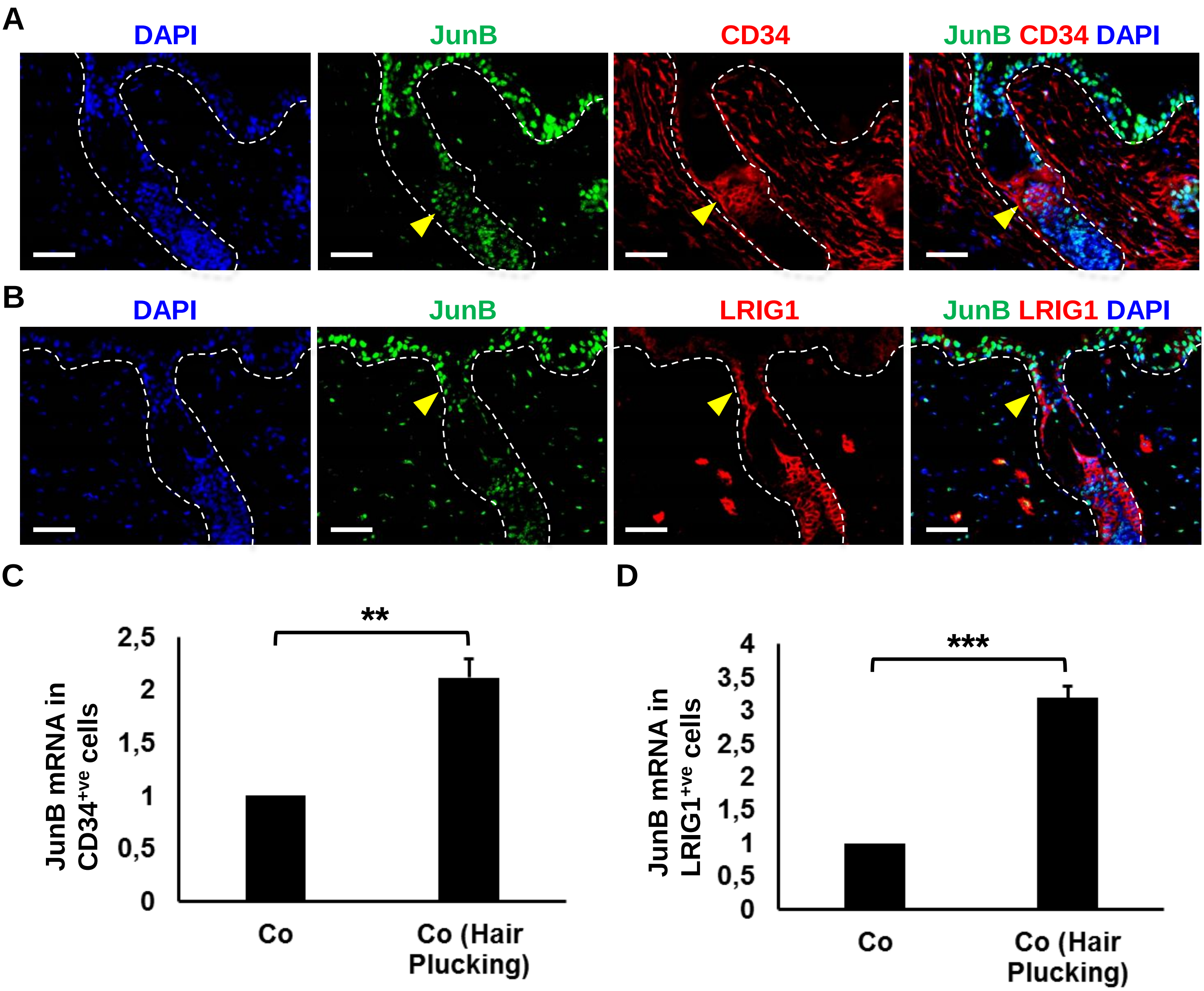

Supplementary Figure 1. Stress stimuli promote JunB expression in skin stem cells.

(A) Co-immunostaining of JunB (green) with the bulge stem cell markers CD34 (red) or **(B)** LRIG1 (red) indicating junctional zone stem cells in the skin of wild type mice at day 4 after hair plucking. Nuclei stained with DAPI in blue. Scale bars, 50 µm. **(C)** Quantification of JunB mRNA in FACS purified $CD34^{+ve}$ and **(D)** $LRIG1^{+ve}$ murine skin stem cells. (n = 3), $^{**}p < 0.01$, $^{***}p < 0.001$, t-test.

Supplementary Figure 2

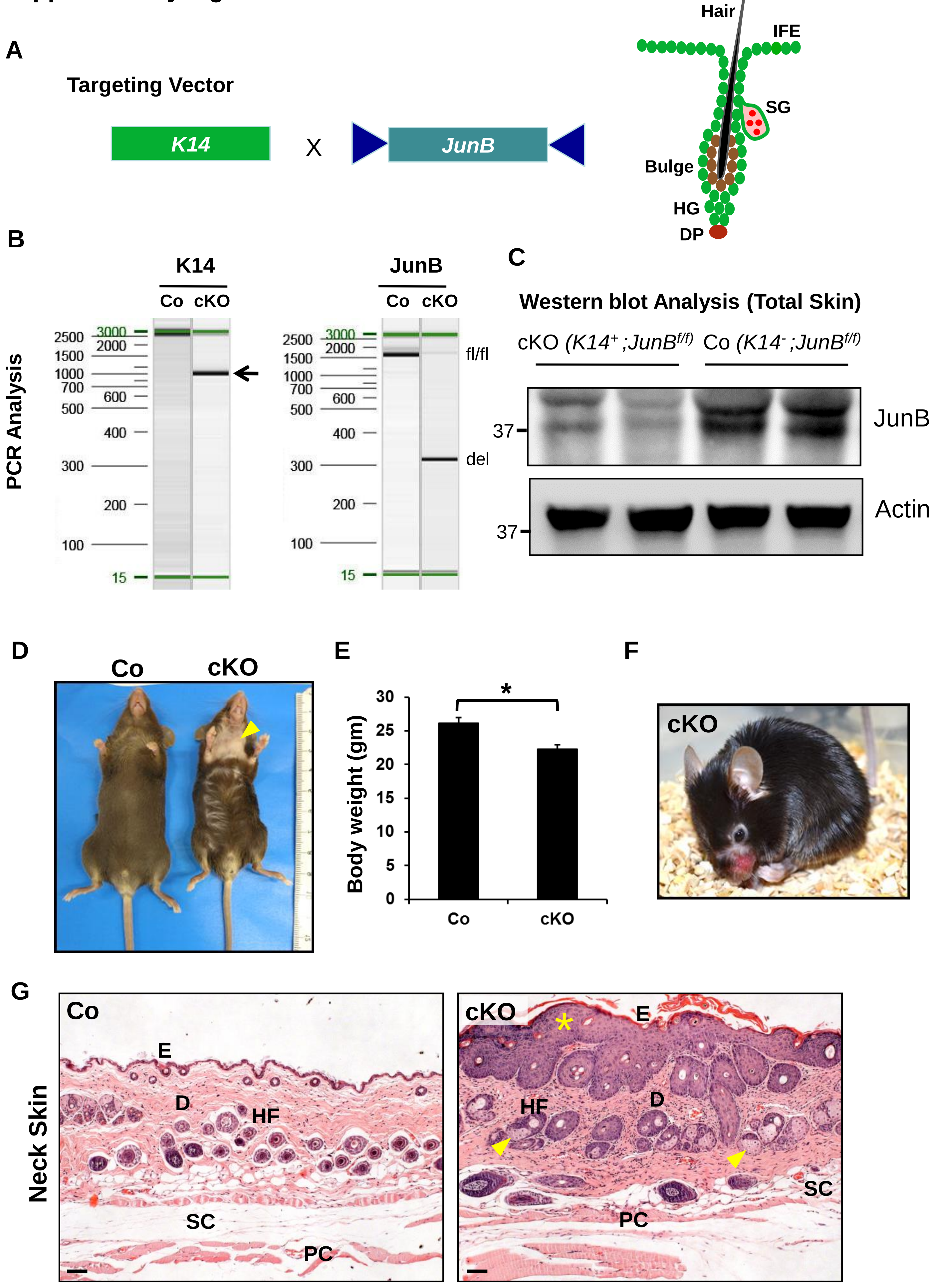

Supplementary Figure 2. JunB cKO mice display reduced body weight and enhanced inflammation.

(A) Cartoon depicting targeting strategy employed to generate basal (K14 promoter) epidermal progenitor cells specific JunB cKO mice. **(B)** PCR results demonstrating successful deletion of JunB in basal epidermal progenitors. **(C)** Western blot analyses confirming JunB deletion in skin. **(D)** Representative clinical picture depicting the abdominal site of 12 weeks old wild type mice (Co) and basal epidermal progenitors-specific JunB cKO mice (cKO). The cKO mice depict alopecia in the upper breast and neck area (yellow triangle) **(E)** Graph displaying body weight of JunB cKO and wild type mice. (n = 5). *p < 0.05, t-test. **(F)** Clinical picture showing itching behavior of JunB cKO mouse with a facial dermatitis-like pathology. **(G)** Representative hematoxylin and eosin (HE) photomicrographs of neck skin from JunB cKO and wild type mice. Note epidermal (asterix) and sebaceous gland hyperplasia (arrow heads) and an inflammatory cellular infiltrate in the neck skin from JunB cKO mirroring human seborrheic dermatitis. Scale bars 50µM. E, epidermis; D, dermis; HF, hair follicle; SG, sebaceous gland SC, subcutaneous layer; PC, panniculus carnosus.

Supplementary Figure 3

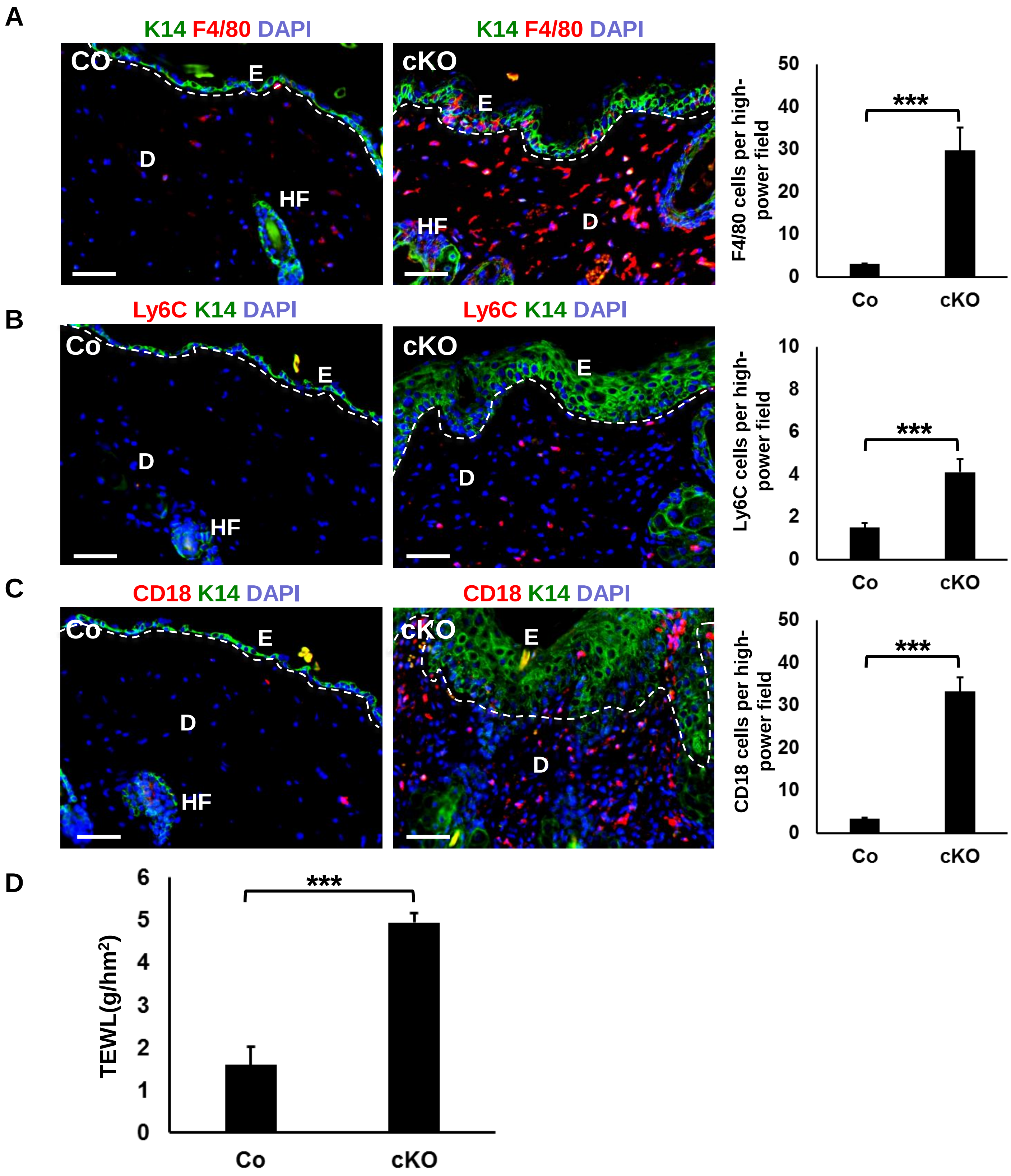

Supplementary Figure 3. Enhanced inflammatory infiltrate in JunB cKO skin.

(A) Immunostaining and quantification of macrophage marker F4/80 (red) and K14 (green) in neck skin from wild type and JunB cKO mice. Nuclei stained with DAPI in blue. Scale bars, 50 µm. ***$p < 0.001$, t-test ($n = 3$). **(B)** Immunostaining and quantification of neutrophil marker Ly6C (red), indicative of neutrophils, and K14 (green) in neck skin from wild type and JunB cKO mice. Nuclei stained with DAPI in blue. Scale bars, 50 µm. ***$p < 0.001$, t-test ($n = 3$). **(C)** Immunostaining and quantification of pan leukocyte marker CD18 (red) and K14 (green) in neck skin from wild type and JunB cKO mice. Nuclei stained with DAPI in blue. Scale bars, 50 µm. ***$p < 0.001$, t-test ($n = 3$). E, epidermis; D, dermis; HF, hair follicle. **(D)** Measurement of TEWL, which is inversely related to epidermal barrier function, on the back skin of JunB cKO mice was significantly enhanced as opposed to wild type mice ($n=3$), ***$p < 0.001$, t-test.

Supplementary Figure 4

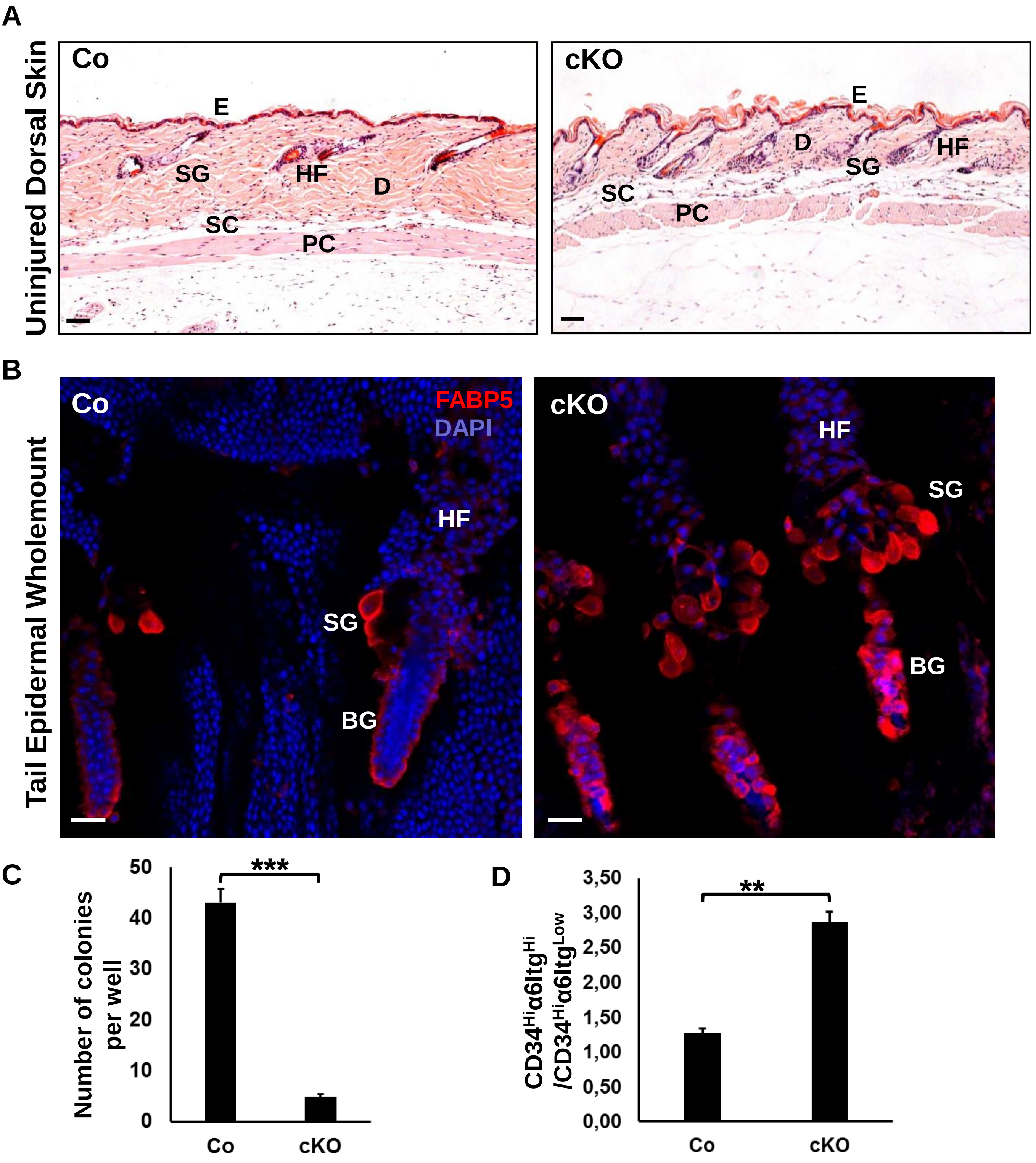

Supplementary Figure 4. JunB regulates differentiation of epidermal stem cells.

(A) Representative H&E microphotographs shows skin histology of unperturbed dorsal skin of JunB cKO and wild type mice. Scale bars, 50 µm. E, epidermis; D, dermis; HF, hair follicle; SG, sebaceous gland; SC, subcutaneous layer; PC, panniculus carnosus. Scale bars, 50 µm. **(B)** Confocal images of whole mount tail epidermis demonstrating enlarged sebaceous glands marked with FABP5 (red) in JunB cKO compared to wild type mice. Nuclei stained with DAPI in blue. Scale bars, 50 µm. **(C)** Quantification of HFSCs colonies depicted in Figure 2C. $^{***}p < 0.001$, t-test ($n = 3$). **(D)** Ratio of undifferentiated (P2) to differentiated (P1) hair follicle stem cell ($CD34^{+ve}\alpha6Itg^{Hi}$ /$CD34^{+ve}\alpha6Itg^{Low}$) indicated in Figure 2D. $^{**}p < 0.01$, t-test ($n = 3$).

Supplementary Figure 5

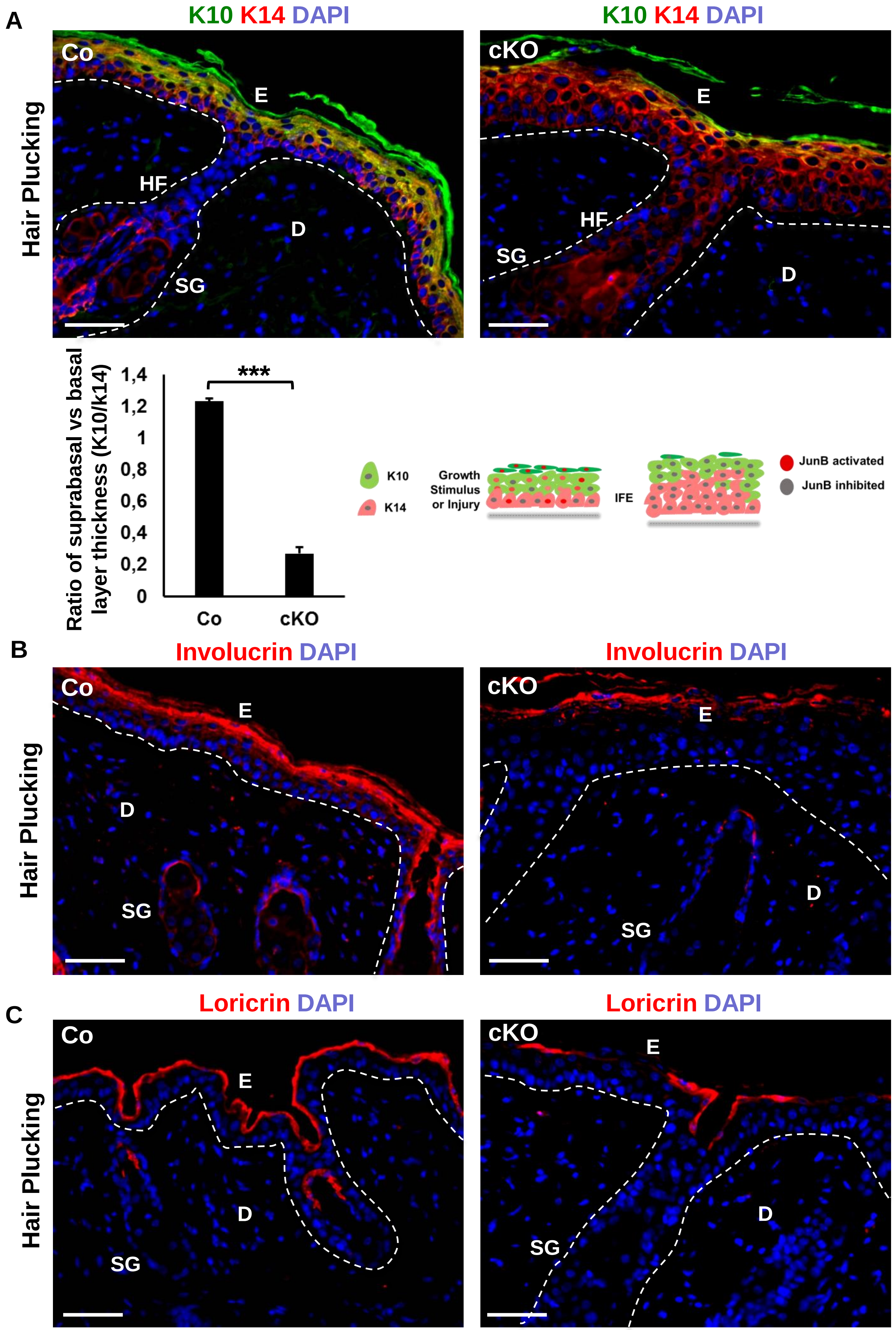

Supplementary Figure 5. Impaired terminal differentiation of epithelial cells in JunB cKO mice.

(A) Representative microphotographs with immunostaining of the keratinocyte differentiation marker K10 (green) and the keratinocyte non-differentiation marker K14 (red), in JunB cKO and wild type mice skin. Nuclei stained with DAPI in blue. Scale bars, 50 µm. The ratio of the suprabasal to basal layers thickness from JunB cKO and wild type mice skin is depicted in graph. ***p < 0.001, t-test (n = 3). **(B)** Immunostaining of late differentiation markers Involucrin (red) and **(C)** Loricrin (red) in the cornified envelope of JunB cKO and wild type mice skin. Nuclei stained with DAPI in blue. Scale bars, 50 µm.

Supplementary Figure 6

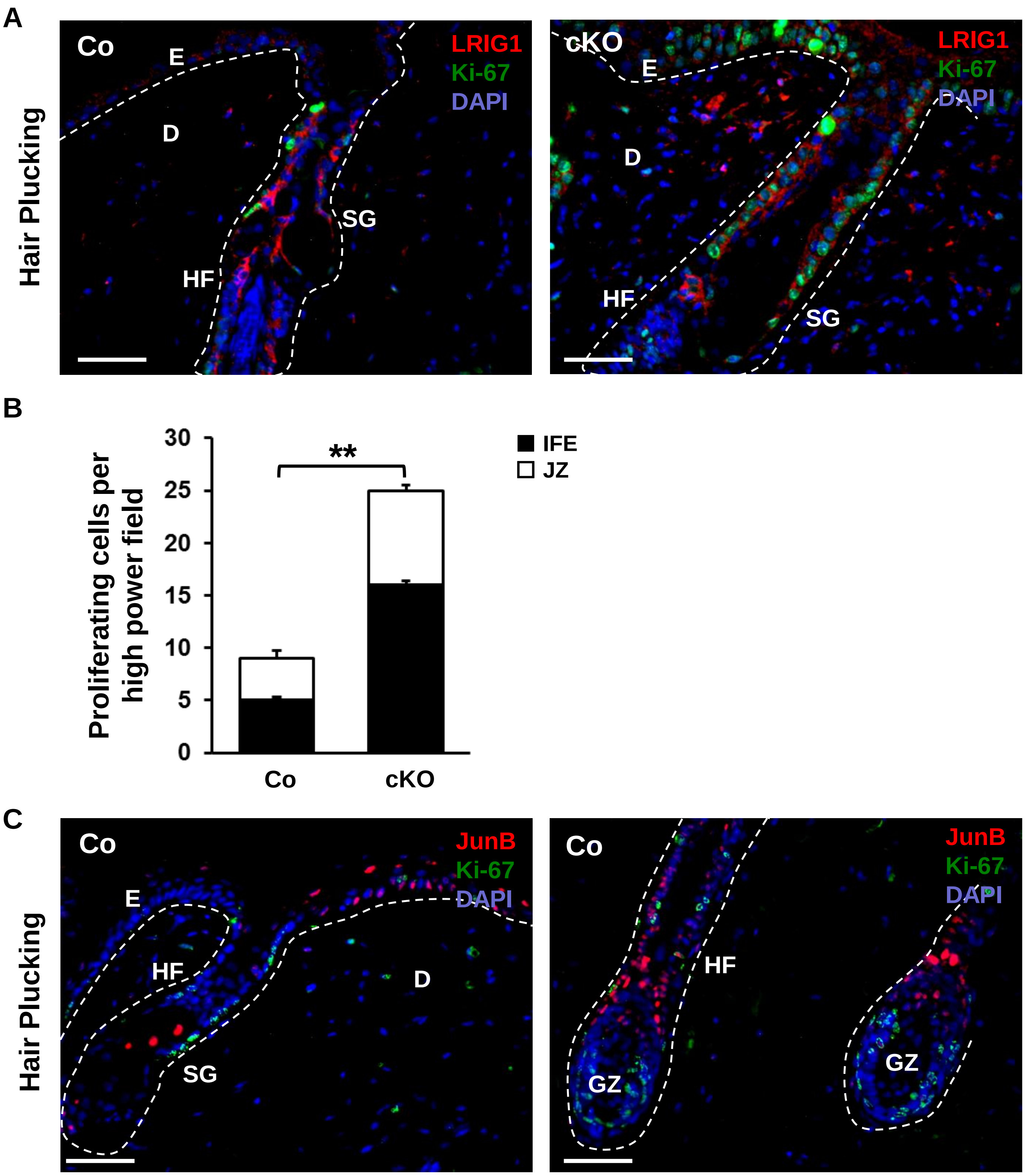

Supplementary Figure 6. JunB restricts hyperproliferation of epidermal progenitors.

(A) Representative photomicrographs of double immunostaining of Ki-67 (green) and LRIG1 (red), indicative of putative sebocyte progenitors. **(B)** Quantitative assessment revealed enhanced proliferation of interfollicular epidermal (IFE) keratinocytes and junctional zone (JZ) sebocyte progenitors marked with LRIG1 following hair plucking-induced growth in JunB cKO compared to wild type skin. **$p < 0.01$, t-test ($n = 3$). **(C)** Double immunostaining of Ki-67 (green) and JunB (red) following proliferation induction triggered by hair plucking in wild type skin. Note that JunB predominantly marks non-proliferating cells. Nuclei stained with DAPI in blue. Scale bars, 50 µm. E, epidermis; D, dermis; HF, hair follicle; SG, sebaceous gland; GZ, growth zone.

Supplementary Figure 7

A

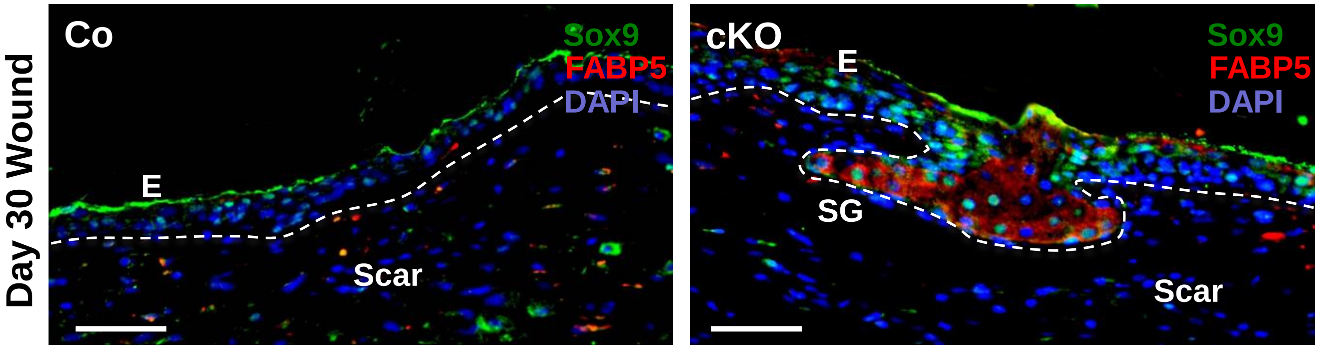

B

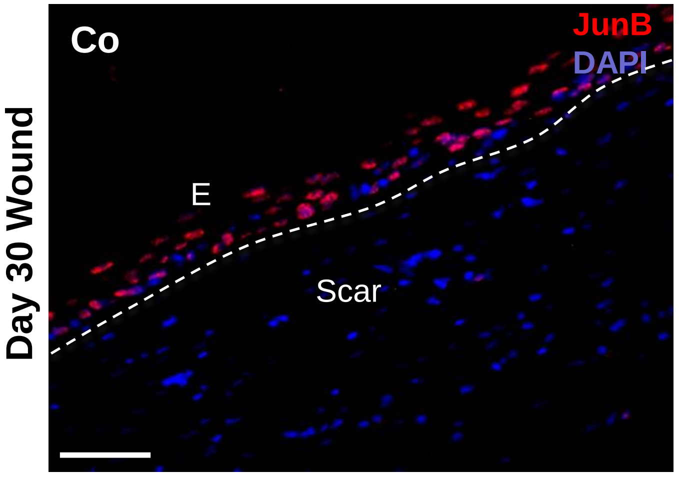

Supplementary Figure 7. Transcription factors maintain skin homeostasis.

(A) Immunostaining of skin for the hair follicle stem cell marker Sox9 (green) and FABP5 (red) marking sebaceous glands in regenerating wound epidermis from wild type and JunB cKO mice. Nuclei stained with DAPI in blue. **(B)** Immunostaining of JunB (red) in epithelial cells residing in regenerating scar epidermis from wild type mice. Nuclei stained with DAPI in blue. E, epidermis; SG, sebaceous gland. Scale bars, 50 µm.

Supplementary Figure 8

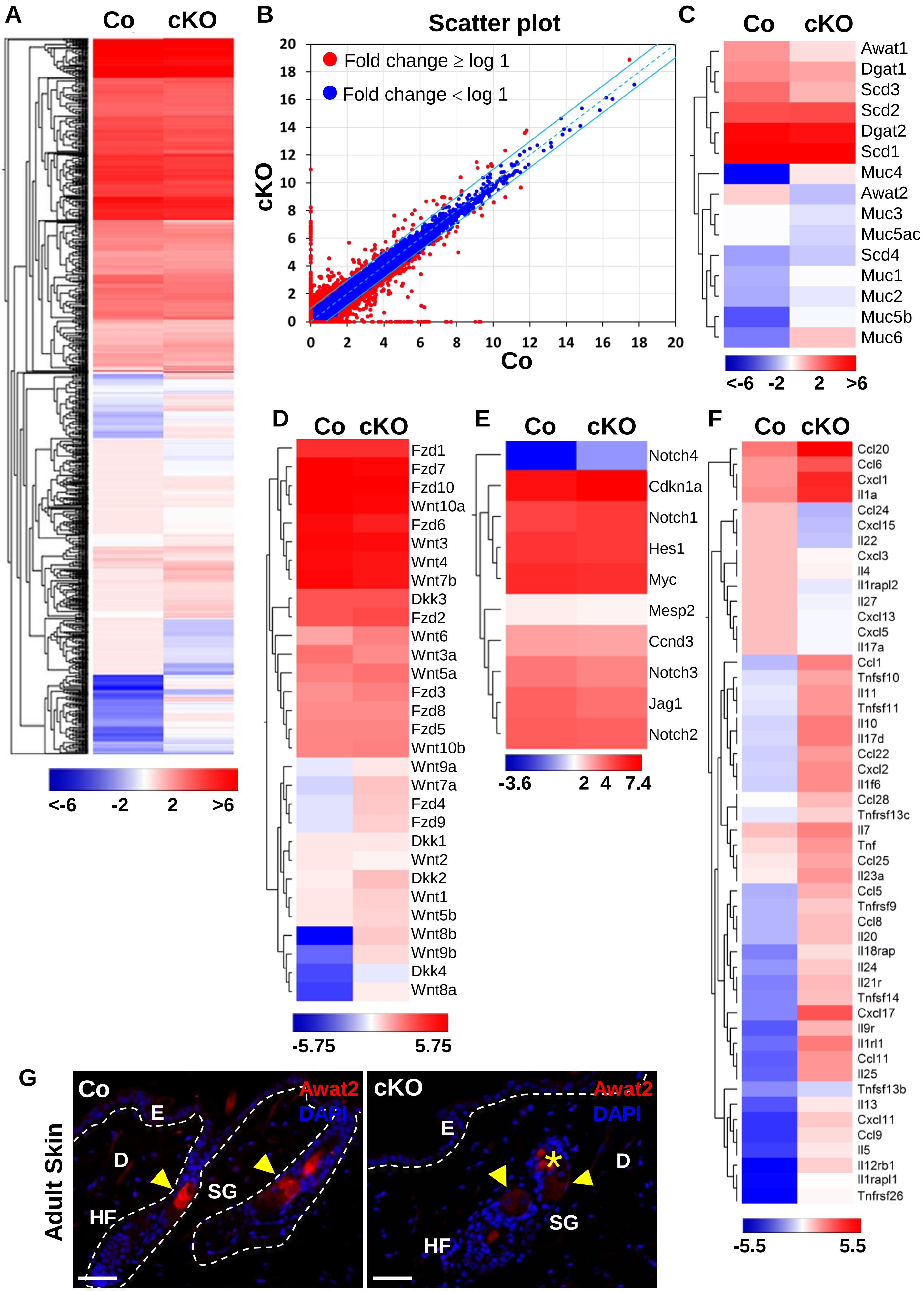

Supplementary Figure 8. JunB deficiency altered global transcriptome in unperturbed skin.

(A) Heatmap depicting transcriptome profiling of samples (n=3) from unperturbed co and JunB cKO skin. The color reflects the log2 scale of relative expression as in the case of Figure (A, C, D, E and F). **(B)** Scatter plot displays the distribution of significantly altered genes derived from independent preparations (n=3) of unperturbed wild type (Co) and JunB cKO skin (cKO). Red color depicts significantly changed genes (2 fold), while blue color denotes unchanged expression of genes. **(C)** Heatmap depicting expression of genes involved in fat metabolism of sebaceous glands, **(D)** Wnt signaling, **(E)** Notch signaling and **(F)** inflammatory processes from co and JunB cKO skin. **(G)** Representative microphotographs with immunostaining of Awat2 (red), an enzyme essential for wax synthesis, in JunB cKO and wild type mice skin. Asterisk indicates hair shaft autofluorescence. Nuclei stained with DAPI in blue. Scale bars, 50 µm. E, epidermis; D, dermis; HF, hair follicle; SG, sebaceous gland.

Supplementary Figure 9

A

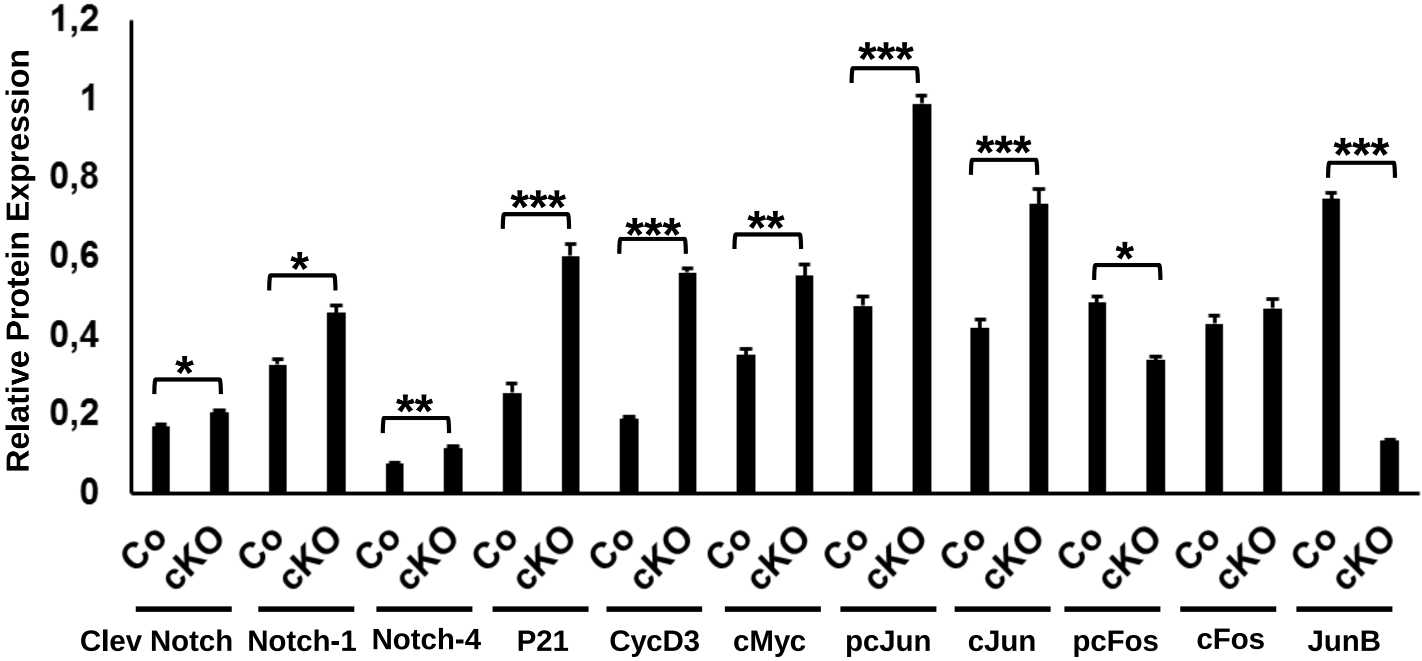

B **ATAC-seq *de novo* motifs in JunB cKO**

High expressed gene sets

Factor	Motif	P-value	% of Targets
E2F	TGCGGCGCGA	1e-2385	0.79%
BORIS(Zf)	CGCCCCCTGGTG	1e-1781	1.73%
Zfx	CCGCGGCTAGCG	1e-1515	0.39%
Sp5(Zf)	GAAGCTCCGCCC	1e-1009	0.29%
AP1	ATGAGTCA	1e-944	1.96%
Zfp161	CCCCGCGCGG	1e-891	1.82%
CCAAT-box	CAGCCAATCA	1e-833	0.31%
CEBP/Sp100	CGACGCGGCGAC	1e-755	0.39%
Klf	CCGGCCACGCCC	1e-595	0.24%
Sp/E2F	CCCGCCTT	1e-298	0.42%

Low expressed gene sets

Factor	Motif	P-value	% of Targets
HINFP/MIZF	CCCGTCCG	1e-12236	4.38%
CTCF	CTGGCCAGCAGG	1e-12022	4.33%
Zic(Zf)	CGAGCCGG	1e-9192	3.59%
SOX9/SOX10	ACAGAGCA	1e-8932	3.52%
AP1	GTGACTCA	1e-8336	5.74%
PRDM1	CTGCCTTCACTT	1e-7893	3.24%
TP53	CATGCCTGTGCA	1e-6363	3.60%
Tcf/GATA	TTAGCTAGCG	1e-5755	5.01%
Smad	GCAGACTG	1e-5210	3.19%
Ets1	CTCACTTCCTGA	1e-3627	3.10%

C **Enrichment of AP1 motifs in ATAC-seq peaks**

Cell type	Motif		P-value	% of Targets
	Forward logo	Reverse logo		
Epidermal stem cell	GATGAGTCAT	ATGACTCATC	1e-5178	15.31%
Hair follicular stem cell	GAATGACGTCAT	ATGACGTCATTC	1e-146	4.73%
Wound tissue	GATGAGTCAT	ATGACTCATC	1e-13162	24.55%

Supplementary Figure 9. AP-1 family transcription factors regulate skin homeostasis.

(A) Quantification of Western blots are highlighted in Figure 6E. *$p < 0.05$, **$p < 0.01$, ***$p < 0.001$, t-test (n = 3). **(B)** *De novo* motif analyses of ATAC-seq peaks in JunB cKO primary epidermal progenitor cells isolated 4 days post hair plucking from skin. The motifs were analyzed in highly expressed gene sets and low expressed gene sets separately. **(C)** Motif analysis depicting substantial enrichment of AP-1 motifs in wound conditions compared to those from homeostatic epidermal and hair follicle stem cells (This analysis was performed using previously deposited ATAC-seq data GEO:GSE89928 from *Ge et al.*).

Supplementary Figure 10

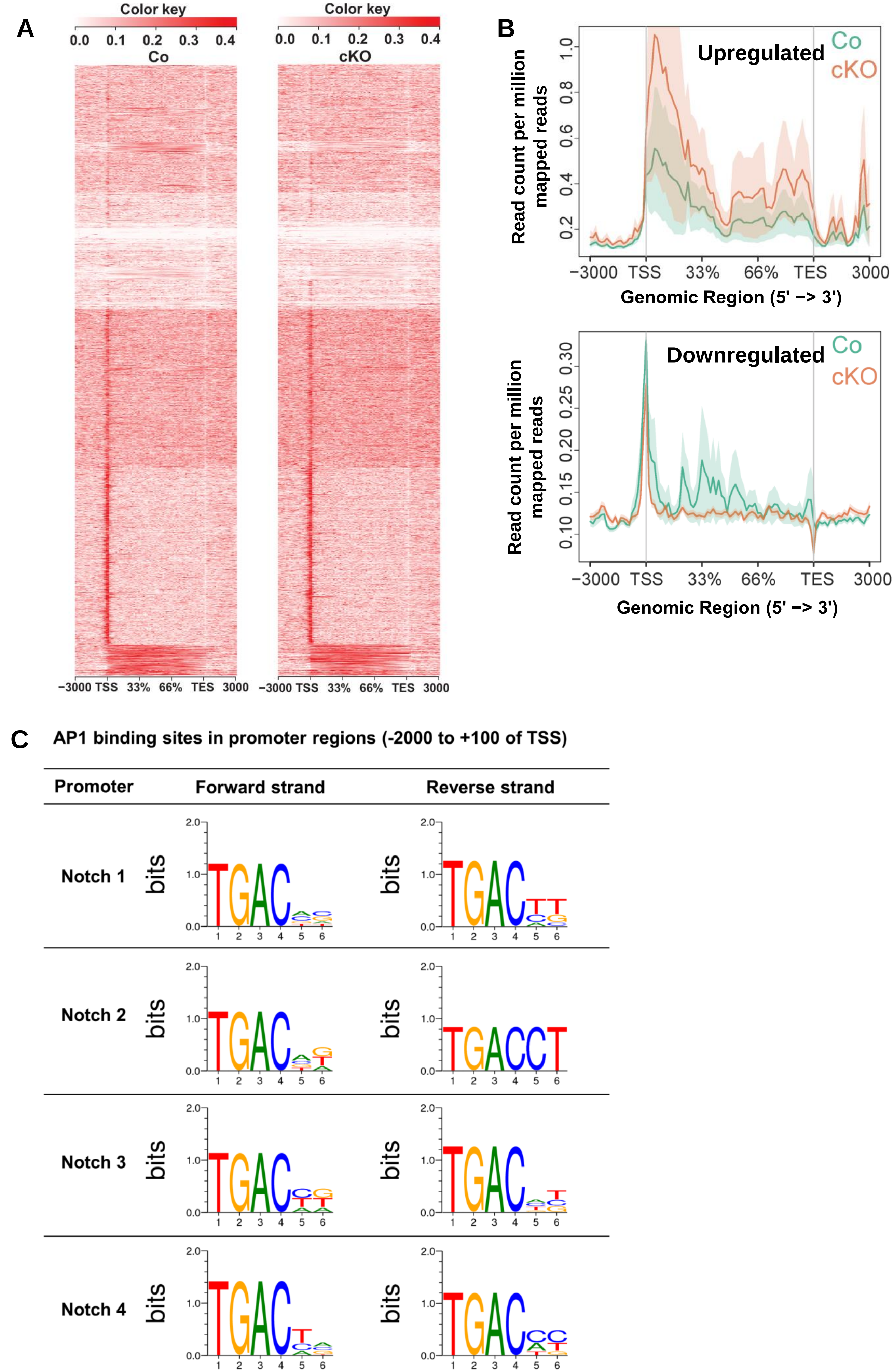

Supplementary Figure 10. JunB directly interacts with promoters of Notch genes.

(A) Heatmap displaying distribution of ATAC-seq signals over genomic region in basal epidermal progenitor cells harvested from wild type and JunB cKO epidermal progenitor cells. **(B)** The distribution of chromatin accessibility (ATAC-seq signals) across the gene body of upregulated and downregulated gene sets in JunB cKO (compared with control) primary epidermal progenitor cells isolated 4 days post hair plucking from skin. **(C)** *In-silico* analysis displays potential JunB binding sites in the promoter region of Notch family genes.

Supplementary Figure 11

A

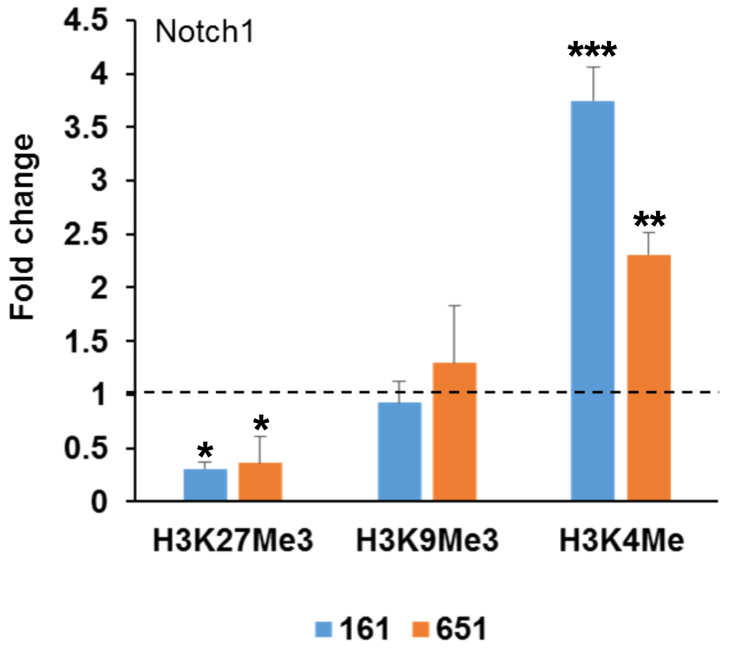

B

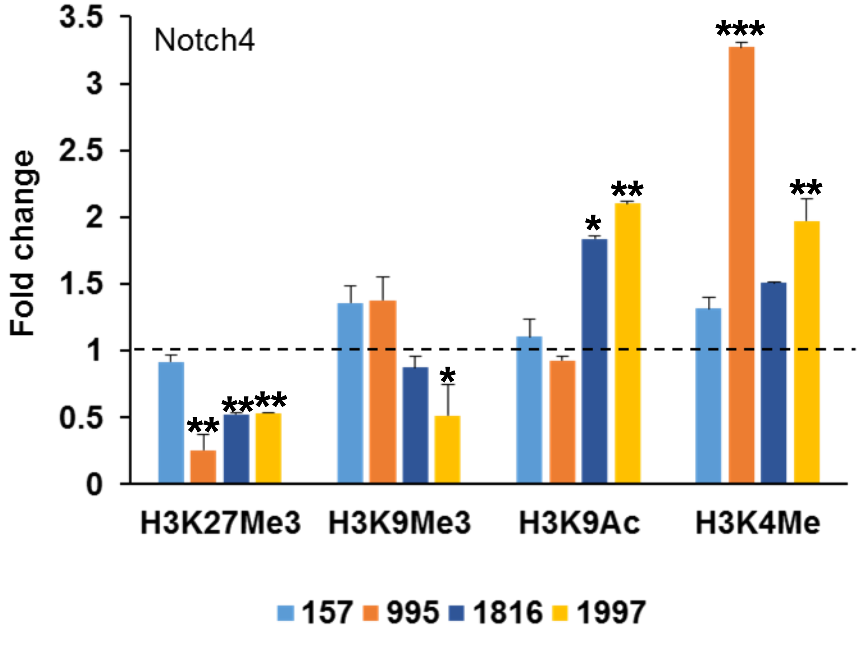

C

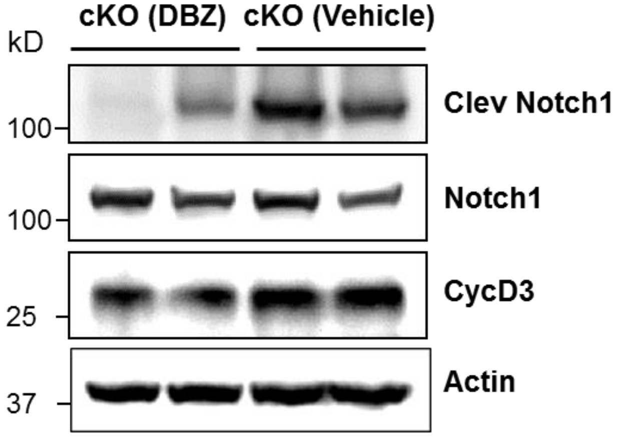

Supplementary Figure 11. JunB act as a repressor of Notch signaling.

(A) ChIP-qPCR assay depicts the association of a reduction in repressive histone marks (H3K27Me3 and H3K9Me3) and a gain of active histone marks (H3K9Ac and H3K4Me) within the promoter region of the Notch1 and **(B)** the Notch4 gene in cKO as opposed to control basal epidermal progenitor cells. $*p < 0.05$, $**p < 0.01$, $***p < 0.001$, t-test ($n = 3$). Bar represents relative position of JunB/AP1 sites in respective promoters. **(C)** Western blot analysis of key Notch pathway proteins in vehicle or Notch inhibitor (DBZ) treated mice skin.

Supplementary Figure 12

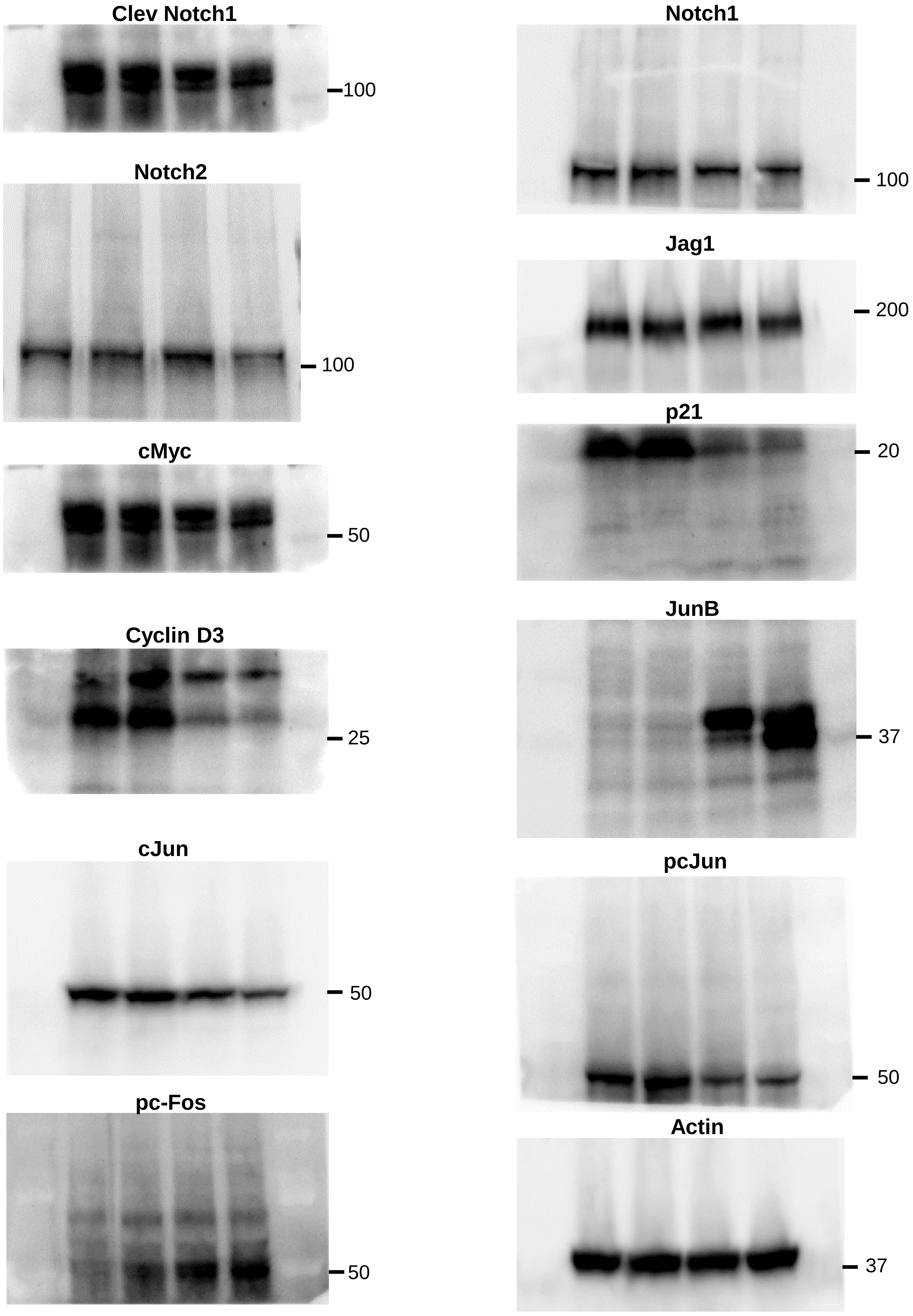

Supplementary Figure 12. Western blot analyses of Notch pathway and AP-1 proteins.

Original uncropped pictures of Western blot analyses presented in Figure 6E. The blots showed the key activated Notch pathway proteins and AP-1 members profiles, 4 days post hair plucking in JunB cKO and wild type skin.

Supplementary table

Supplementary Table 1: Summary of the LC-MS results.

RT	Annotations	Elemental formula	[M+H]+	Parent ion	Product ions			Neutral loss
**4.846**	Palmitoyl-EA	C18H37NO2	300.2897	**300**		282	62	**18**
**5.613**	Oleoyl-EA	C20H39NO2	326.3054	**326**		308	62	**18**
**7.135**	Stearoyl-EA	C20H41NO2	328.3210	**328**		310	62	**18**
**14.31**	EA-C34H67NO3	C34H67NO3	538.5194	**538**	300	282	62	**238**
**14.52**	EA-C36H69NO3	C36H69NO3	564.5350	**564**	328	310	62	**236**
**14.64**	EA-C35H69NO3	C35H69NO3	552.5350	**552**	300	282	62	**252**
**14.75**	EA-C38H71NO3	C38H71NO3	590.5507	**590**	326	308	62	**264**
**14.99**	EA-C36H71NO3	C36H71NO3	566.5507	**566**	300	282	62	**266**
**14.99**	EA-C36H71NO3	C36H71NO3	566.5507	**566**	328	310	62	**238**
**15.18**	EA-C38H73NO3	C38H73NO3	592.5663	**592**	326	308	62	**266**
**15.18**	EA-C38H73NO3	C38H73NO3	592.5663	**592**	328	310	62	**264**
**15.59**	EA-C38H75NO3	C38H75NO3	594.5819	**594**	328	310	62	**266**

Supplementary Table 2: Primers list

**Name**	**Sequence**
**Genotyping**	
K14 Cre1	CAATTTACTGACCGTACA
K14 Cre2	TAATCGCCATCTTCCAGCAG
JunB-B1	ATCCTGCTGGGAGCGGGGAACTGA
JunB-B6	AGAGTCGTCGTGATAGAAAGGC
**qPCR**	
JunB_FP	TTTCTATCACGACGACTCTTACGCAGC
JunB_RP	AAGGTGGGTTTCAGGAGTTTGTAGTCG
Actin_FP	CCTTCTTGGGTATGGAATCCTGTGG
Actin_RP	CAGCACTGTGTTGGCATAGAGGTCTTTAC

Supplementary Table 3: ChIP primers

Notch1_161_FP1	GTGGTTACAATGTTACAGGTCTATCAGC
Notch1_161_RP1	CACACACCAAAACCAAAGCTGTC
Notch1_584_635_FP2	ATGGCAGGTAGGCAGATGTC
Notch1_584_635_RP2	GGCAGCCTGACTTGGATACAAAG
Notch1_651_FP3	GGCTGCCTGCTGACAAAG
Notch1_651_RP3	TTCTGAGATGGGTCCTGAAGGC
Notch1_723_FP4	GACACATAAGCAGTTAGAGGCCTTC
Notch1_723_RP4	TCCAGAGAGCAGTTCCATAGAAGG
Notch1_3‘_UTR_FP	CTTCATGTCCTGAGCTGGGATC
Notch1_3‘_UTR_RP	TTCCTCCTGCATCTACCCCTTC
Notch4_157_FP1	CCAAAACACCTCAAACATCAGACAG
Notch4_157_RP1	TTAGCCTTATATTGGGAATTTAGTCAGC
Notch4_477_FP2	TAGCAATTAGGAGATGGAAGCAGG
Notch4_477_RP2	GGCCTTGAACTTGCTGTGTTG
Notch4_995_1014_1026_FP5	GGTAATGAATTCCCTTTATGGTTGTC
Notch4_995_1014_1026_RP5	GGGTACAGTCAAGATGTTAGTCAAG
Notch4_1648_FP9	AAGAGCCAAGGAAAGGTCTAAAGATC
Notch4_1648_RP9	GCAGCAACGTGTCTTGAACTG
Notch4_1816_1828_1851_FP12	CTGTGAAAAGGAAGCCGTATTG
Notch4_1816_1828_1851_RP12	CTCTTAGGGCCCAGGGTG
Notch4_1997_FP13	CCATCAGTAGGGTGTCCAGG
Notch4_1997_RP13	CTACCGCCCTTCTTCCTCC
Notch4_5‘_UTR_FP	TTGGCAGGAGAGACGGTGAG
Notch4_5'_UTR_RP	TGGAGGCAGCTCAGAGCTC
