# Supplementary material for: JunB defines functional and structural integrity of the epidermo-pilosebaceous unit in the skin
Source: Nat Commun. 2018 Aug 24;9:3425. doi: 10.1038/s41467-018-05726-z (PMC6109099; doi:10.1038/s41467-018-05726-z)
Supplement: Supplementary file 2 — Peer Revies File [file 41467_2018_5726_MOESM2_ESM.pdf]

Reviewers' comments:

Reviewer #1 (Remarks to the Author):

This manuscript by Singh et al., reveals novel functions for JunB in murine skin using a transgenic mouse model in which K5 promoter driven Cre recombinase is used to delete JunB in an epidermal-specific manner. Although a similar approach has been taken previously using the K14 promoter (Meixner et al., 2008; Pflegerl et al., 2009), the resulting defects in these mice appear to have some commonalities i.e. hyperproliferation, skin lesions and an inflammatory and proliferative response to stimuli such as TPA. However, an important and distinct phenotype to that previously reported is shown here centred around effects on sebaceous glands. However, I have some reservations around the data on enlargement of sebaceous glands in the JunB deficient mice shown in Fig 2E - the tissue was taken from the neck region which may be activated by scratching in these mice that appear to suffer from itching and moreover appears to show hyperproliferation in all the epithelium. Skin tissue from other relatively unperturbed sites e.g. the dorsal skin would be useful. The most impressive observation in the entire manuscript is that de novo sebaceous glands are generated 30 days post-wounding apparently derived from the neo-epidermis suggesting that JunB may be involved in restricting differentiation to the interfollicular epidermal lineage. This part of the study represents a novel advance to our current understanding of the potential role of this transcription factor in skin biology. However, the remaining data and conclusions drawn from it are perhaps overstated given their descriptive nature and the absence of any functional evidence.

The alterations in transcriptional profile do indeed indicate that the system is perturbed, but the extent to which the authors claim that epidermal functions are altered from the lipid analysis is not supported by the data presented. For instance, an altered lipid profile performed only once from hairs rather than sebaceous glands without replicates and unaccompanied by any kind of functional assay or correlation with human disease dampens enthusiasm. The claim that epidermal barrier is affected in mutant mice particularly in non-challenged skin is not substantiated by functional evidence. Aside from K10, the authors show no analysis of differentiation-related proteins that form part of the cornified envelope to support their argument.

The observation that wounding is delayed in spite of increased cellularity is also of interest but not exploited sufficiently to gain a mechanistic understanding. Moreover, in order to claim that the injured epithelium is hyperproliferative requires comparison with uninjured mutant skin not WT controls as shown in Figure 5 B-D and Figure 6 A.

The claim that junB may affect epidermal differentiation needs to be substantiated with evidence that this factor actually binds to the Notch gene promoter via ChIP-seq analysis and evidence for loss of signalling through this pathway in mutant skin, the in silico for potential binding sites not withstanding.

The authors seem unclear about the origin of the sebaceous gland cells in wounded tissue – implying that they may come from Sox-9 positive hair follicle stem cells while at the same time conjecturing that they arise de novo from epidermal progenitors due to de-repression of the sebocyte lineage. Have they examined whether the sebaceous glands arising during wound healing in the mutant mice eventually also regenerate entire hair follicles?

The authors should include a comprehensive transcriptional profile of the mutant skin so that a comparison with existing data on other knockout mice in the AP-1 family can be compared.

Reviewer #2 (Remarks to the Author):

Singh et al investigate the role of JunB/AP-1 in epidermal homeostasis and inflammation, with a focus on sebaceous gland pathology. This manuscript replicates previous findings showing that JunB deletion in the epidermis leads to aberrant skin homeostasis and inflammation (Meixner et al., 2008). The results showing abnormal sebaceous glands in these mice are novel; however, the findings are mostly descriptive with no mechanistic insights. Thus, this manuscript is not appropriate for publication in Nature Communications.

Major points:
1) A major part of the abstract is a repetition of the previously published findings. The authors have interesting data regarding the sebaceous gland pathology and lipid profile in these mice, thus, they should make an effort to focus on these findings, expand on the mechanistic regulation and remove the previously published work, since it is distracting and not necessary. Many of the findings described as novel are already in the previous paper.
2) Figures 1 and 2 do not contain any new information (except for the EM pictures) and thus should be moved to Supplementary Figures.
3) Many conclusions in this manuscript are not supported by data. Examples are below:
i) In Figure 1, the authors claim that JunB expression is upregulated in "undifferentiated basal keratinocytes, more defined hair follicle stem cells including LRIG1 and CD34 positive populations" upon TPA challenge. These conclusions are not well demonstrated in Figure 1C, D or E. For example, the IF in Fig1E showing double staining of JunB and CD34 or LRIG1 is not clear. The authors should show single color images and corroborate these findings with another method (for example sort these cells by FACS and determine mRNA expression levels of JunB in different populations).
ii) In Figure 2, the authors claim that "suprabasal" JunB is responsible for the suppression of inflammation in the skin. Which experiments demonstrate this claim?
iii) In Figure 2, the authors claim that the JunB mutant mice have epidermal barrier defects. Again, there are no experiments to address this claim.
iv) In Figure 4, the authors indicate that the differentiation of hair follicle stem cells is impaired in JunB mutant skin. To make such a conclusion, the authors have to carry out further experiments, such as double staining of CD34 with FABP5 and LRIG1 with FABP5. Also, expression analyses of Sox9 and Keratin 15, as well as stemness and quiescence markers are needed.
v) In Figure 4B, the authors investigate the ratio of CD34 subpopulations with a6integrin and conclude that the differences observed indicate defects in maintaining stem cell homeostasis. Which part of the mouse was used for these experiments? Inflamed skin or tail skin? To make such a conclusion, in vitro clonogenic assays are needed. The small differences shown in the dot plots are not sufficient to make such a claim.
4) In Figure 1 and 2 more details should be provided, such as age of mice at analysis, age when disease is complete, area of skin analyzed etc.
5) In Figure 4A, FABP5 expression is observed not only in the sebaceous gland but also in the outer layer of the epidermis. The authors should comment on that finding. To demonstrate that FABP5 is expressed in the bulge, co-staining with CD34 or Keratin 15 is necessary.
6) The authors show that the promoters of Notch family genes have AP-1 binding sites. AP-1 family functions are dependent on dimer composition etc. and are very complex. To determine if AP-1 and in this case specifically JunB actually transcriptionally regulates Notch, Chromatin IP experiments are needed.
7) The authors show changes in many different pathways in JunB mutant skin through both RNA-seq and target gene analysis, such as lipids, cholesterol, Notch pathway etc. To determine whether any of these alterations are functionally relevant, rescue experiments should be performed.

Minor comments:

1) It is recommended to improve the compensation in the dotplots in Figure 4B.
2) Markers used to identify populations such as sebaceous glands should be more consistent throughout the manuscript, e.g. FABP5 in Figure 4 and SCD1 in Figure 6.
3) In the discussion the authors comment that the sebaceous gland findings were not documented

in Zenz et al., 2005. This is a completely different mouse model, and the JunB deletion in Keratin-5 expressing tissues was published by Meixner et al. The discussion should be changed accordingly.

We appreciate the editor´s and reviewers´ constructive review of the above mentioned manuscript. The whole manuscript has been thoroughly revised and amended, including new data from the experiments suggested by the editor and reviewers. The relevant references were also cited and added to the reference list.

Reviewer 1

This manuscript by Singh et al., reveals novel functions for JunB in murine skin using a transgenic mouse model in which K14 promoter driven Cre recombinase is used to delete JunB in an epidermal-specific manner. Although a similar approach has been taken previously using the K5 promoter (Meixner et al., 2008; Pflegerl et al., 2009), the resulting defects in these mice appear to have some commonalities i.e. hyperproliferation, skin lesions and an inflammatory and proliferative response to stimuli such as TPA. However, an important and distinct phenotype to that previously reported is shown here centered around effects on sebaceous glands.

Re 1a: However, I have some reservations around the data on enlargement of sebaceous glands in the JunB deficient mice shown in Figure 2E - the tissue was taken from the neck region which may be activated by scratching in these mice that appear to suffer from itching and moreover appears to show hyperproliferation in all the epithelium. Skin tissue from other relatively unperturbed sites e.g. the dorsal skin would be useful.

Reply: This is a valid concern raised by Reviewer 1. We now have incorporated representative photomicrographs showing immunostaining for the sebaceous gland marker FABP5 **(Figure 2A and S4B)** and H&E stained sections **(Figure 2B and S4A)** from unperturbed skin of the back and tail. Independent of the neck skin which – due to scratching may reveal sebaceous gland hyperplasia, we also found a substantial enlargement of sebaceous glands in JunB mutant mice in unperturbed skin regions. These data imply that JunB deficiency in basal keratinocytes including stem cells is responsible for the observed phenotype of sebaceous gland hyperplasia. Nevertheless, scratching, hair plucking, TPA treatment or wounding with subsequent release of growth factors, further enhance the size of sebaceous glands in JunB mutant mice.

Re 1b: The most impressive observation in the entire manuscript is that de novo sebaceous glands are generated 30 days post-wounding apparently derived from the neo-epidermis suggesting that JunB may be involved in restricting differentiation to the interfollicular epidermal lineage. This part of the study represents a novel advance to our current understanding of the potential role of this transcription factor in

skin biology. However, the remaining data and conclusions drawn from it are perhaps overstated given their descriptive nature and the absence of any functional evidence.

Reply: We are grateful to Reviewer 1 for this most constructive comment. According to her/his valuable suggestion, we undertook significant effort to increase the mechanistic insight into our finding that JunB is important to suppress lineage plasticity. Based on a global RNA-seq approach of skin perturbed by wounding or hair plucking and subsequent pathway analyses, we found that Notch signaling is activated among other pathways **(Figure 6A-6D)**. This was confirmed by Western blot analysis depicting Notch target genes to be highly up-regulated in JunB mutant skin **(Figure 6E)**. In addition, ChIP-Seq experiments showed that JunB, indeed, binds to regions in the Notch 1 and Notch 4 promoter (**Figure 6H and 6I**). Most importantly, employing the Notch inhibitor DBZ, the generation of sebaceous glands in the neo-epidermis of 30 days old wounds was completely suppressed **(Figure 7)**. These data mechanistically imply that JunB is involved in restricting differentiation to the interfollicular epidermal lineage and, thus, suppress lineage plasticity.

In addition, following this Reviewer´s advice, we concentrated on this novel aspect of our findings in the revised manuscript, and transferred less important, descriptive data to the supplements or even omitted them.

Re 1c: The alterations in transcriptional profile do indeed indicate that the system is perturbed, but the extent to which the authors claim that epidermal functions are altered from the lipid analysis is not supported by the data presented. For instance, an altered lipid profile performed only once from hairs rather than sebaceous glands without replicates and unaccompanied by any kind of functional assay or correlation with human disease dampens enthusiasm.

Reply: We addressed the concerns of this Reviewer as follows:

In case of lipid profiling from hairs, we have used at least 3 mice for each subgroup from control or mutated mice, respectively, and the corresponding pooled samples. The experimental groups are shown in the PCA plot **(Figure 5B)**. The clustering of averaged groups is reported in the **Figure 5D**. Due to technical limitation in isolation and purification of fragile sebaceous glands from skin in sufficient quantity for LC-MS analyses, we have analysed sebum deposited on the hair surface. We further wished to correlate changes in lipid components and composition to a defective epidermal barrier. Though indirectly, we showed that JunB mutant skin displayed an increased number of macrophages in the interfollicular epidermis, most likely reflecting the epidermal barrier defects **(Figure S3A)**. In addition, in the revised manuscript, we added data on transepidermal water loss (TEWL) for evaluation of the epidermal barrier function in JunB mutant skin using a TEWA meter. Interestingly, enhanced epidermal water loss indicating poor barrier function in JunB mutant skin was found when compared to control skin **(Figure S3D)**.

Re 1d: The claim that epidermal barrier is affected in mutant mice particularly in non-challenged skin is not substantiated by functional evidence.

Reply: Reviewer 1 is right. In the revised version, as mentioned above **(Re 1c)**, we included the functional assay to determine transepidermal water loss (TEWL). TEWL is inversely related to skin barrier function. We assessed transepidermal water loss (TEWL) in JunB mutant skin using the TEWA meter. We found significantly enhanced epidermal water loss indicating poor epidermal barrier function in JunB mutant skin compared to control skin **(Figure S3D)**.

Re 1e: Aside from K10, the authors show no analysis of differentiation-related proteins that form part of the cornified envelope to support their argument.

Reply: As suggested by Reviewer 1, we now included immunostaining for additional terminal differentiation related markers such as Loricrin and Involucrin confined to the cornified skin envelope **(Figure S5B and S5C)**. These findings are consistent with our earlier observation **(Figure S5A)**.

Re 1f: The observation that wounding is delayed in spite of increased cellularity is also of interest but not exploited sufficiently to gain a mechanistic understanding. Moreover, in order to claim that the injured epithelium is hyperproliferative requires comparison with uninjured mutant skin not WT controls as shown in Figure 5 B-D and Figure 6 A.

Reply: This is again a valid concern raised by Reviewer 1. Delayed wound healing in JunB mutant mice is due to both impaired differentiation and most likely to excessive macrophage dominated inflammation **(Figure S3A)**. Persisting high numbers of macrophages largely contribute to increased cellularity in wounds of JunB mutant mice. In addition, whole transcriptome analyses of JunB wounds revealed evidence for an increased expression of pro-inflammatory cytokines **(Figure 6A and 6C)**. This leads to prolongation of the pro-inflammatory vicious cycle during wound healing, and, in consequence, to delayed wound closure.

As suggested, we now included uninjured age matched dorsal skin from JunB mutant and wild type mice in the manuscript **(Figure S4A)**.

Re 1g: The claim that JunB may affect epidermal differentiation needs to be substantiated with evidence that this factor actually binds to the Notch gene promoter via ChIP-seq analysis and evidence for loss of signalling through this pathway in mutant skin, the in silico for potential binding sites notwithstanding.

Reply: This excellent advice from Reviewer 1 substantially helped us to improve the quality of manuscript. To systematically investigate the impact of JunB loss on Notch signaling, we have performed whole transcriptomic analyses in hair depilated and wounded skin **(Figure 6A, 6B and 6C)**. Intriguingly, our analyses revealed significant up-regulation in Notch signaling among other pathways under both stress conditions **(Figure 6A, 6B and 6C)**. We found Notch is particularly interesting as this is a major pathway regulating proliferation and differentiation in skin. Consistent with these findings, we observed a marked activation of Notch receptors and their target genes such as p21, CyD3 and cMyc at the protein level. In

addition, expression of other AP-1 members, like cJun changed in the JunB mutants. This possibly, may compensate some but not all phenotypes of JunB mutant mice **(Figure 6E)**. Furthermore, our analyses depicted a marked enrichment of ATAC-seq signals at transcript start sites (TSSs) in JunB mutant as opposed to control **(Figure 6F and S10A)**, suggesting global rewiring of the genome in case of JunB loss.
In addition to *in-silico* analysis **(Figure S10B)**, we also performed ChIP analysis to determine direct physical interaction between JunB and Notch promoters in control basal keratinocytes following hair plucking under *in vivo* conditions. ChIP analysis confirmed multiple binding sites for JunB in the promoter region of Notch 1 **(Figure 6H)**, and Notch 4 gene **(Figure 6I)**, indicating direct regulation of Notch signaling by JunB in the epidermis. This physical interaction is lost due to JunB deficiency, and this may be responsible for the observed changes in the Notch signaling **(Figure 6E)**
In aggregate, our findings suggest that JunB deficiency, though partly inducing compensatory changes in the AP-1 complex, deregulate several pathways including Notch signaling and subsequently may impair epidermal homeostasis.

Re 1h: The authors seem unclear about the origin of the sebaceous gland cells in wounded tissue – implying that they may come from Sox-9 positive hair follicle stem cells while at the same time conjecturing that they arise *de novo* from epidermal progenitors due to de-repression of the sebocyte lineage. Have they examined whether the sebaceous glands arising during wound healing in the mutant mice eventually also regenerate entire hair follicles?
Reply: This is an interesting suggestion raised by Reviewer 1. To explore whether sebaceous glands arising during wound healing in mutant mice eventually also regenerate entire hair follicles, we followed JunB mutant wounds for 90 days. Interestingly, after 90 days after wounding, *de novo* sebaceous glands further elongated and transformed into a tube shaped duct. However, these tube shaped *de novo*-formed sebaceous glands are devoid of hairs, the epithelial root sheet surrounding hairs and any other skin appendages (eccrine and apocrine glands) **(Figure 4D)**. These data indicate that JunB specifically suppress lineage plasticity of basal keratinocytes including stem cells towards ectopic sebaceous glands.

Re 1i: The authors should include a comprehensive transcriptional profile of the mutant skin so that a comparison with existing data on other knockout mice in the AP-1 family can be compared.
Reply: As suggested by the Reviewer 1, a comprehensive transcriptional profile of the mutant skin has been included in the revised manuscript **(Figure 6A-6C and S8A-S8D).**

Reviewer 2

Singh et al investigate the role of JunB/AP-1 in epidermal homeostasis and inflammation, with a focus on sebaceous gland pathology. This manuscript replicates previous findings showing that JunB deletion in the epidermis leads to aberrant skin homeostasis and inflammation (Meixner et al., 2008). The results

showing abnormal sebaceous glands in these mice are novel; however, the findings are mostly descriptive with no mechanistic insights. Thus, this manuscript is not appropriate for publication in Nature Communications.

Re 2a: A major part of the abstract is a repetition of the previously published findings. The authors have interesting data regarding the sebaceous gland pathology and lipid profile in these mice, thus, they should make an effort to focus on these findings, expand on the mechanistic regulation and remove the previously published work, since it is distracting and not necessary. Many of the findings described as novel are already in the previous paper.

Reply: We thank Reviewer 2 for her/his essential suggestion. We revised our manuscript according to her/his suggestions. In detail, we concentrated on getting mechanistic insight into the causal contribution of JunB-dependent Notch inhibition suppressing lineage plasticity in the epidermis. This is an important new aspect of a previously unreported role of JunB in skin biology. We have answered the concern of Reviewer 2 in more detail in the point-to-point answer to Reviewer1 (**R1b)**.

Re 2b: Figures 1 and 2 do not contain any new information (except for the EM pictures) and thus should be moved to Supplementary Figures.

Reply: As suggested, we have moved Figure 2 to the Supplementary Figures, while keeping Figure 1 as JunB expression in the sebaceous glands has previously not been reported. In addition, upregulated of JunB expression in an undifferentiated epidermal stem cell population upon stress signals have not been documented in earlier publications.

Re 2c: Many conclusions in this manuscript are not supported by data. Examples are below: **i)** In Figure 1, the authors claim that JunB expression is upregulated in “undifferentiated basal keratinocytes, more defined hair follicle stem cells including LRIG1 and CD34 positive populations” upon TPA challenge. These conclusions are not well demonstrated in Figure 1C, D or E. For example, the IF in Figure 1E showing double staining of JunB and CD34 or LRIG1 is not clear. The authors should show single color images and corroborate these findings with another method (for example sort these cells by FACS and determine mRNA expression levels of JunB in different populations).

Reply: We are grateful to Reviewer 2 for this specific suggestion which is meant to improve the quality of our manuscript. We have now incorporated single color images in the manuscript **(Figure S1A and S1B)** depicting higher expression of JunB in two distinct epidermal stem cell populations. These findings were further supported by qPCR demonstrating higher abundance of JunB mRNA in FACS purified $CD34^{+ve}$ and $LRIG1^{+ve}$ stem cells upon hair plucking **(Figure S1C and S1D)**.

ii) In Figure 2, the authors claim that "suprabasal" JunB is responsible for the suppression of inflammation in the skin. Which experiments demonstrate this claim?

Reply: This is a valid concern raised by Reviewer 2. These conclusions were drawn from the observations suggesting marked inflammation **(Figure S2G and S3A-C)** and upregulation of pro-inflammatory genes **(Figure 6C)** in JunB mutant skin as opposed to wild type.

iii) In Figure 2, the authors claim that the JunB mutant mice have epidermal barrier defects. Again, there are no experiments to address this claim.

Reply: Reviewer 2 is right, this functional piece of evidence for a disruption of the epidermal barrier function in JunB mutant mice was missing in our first manuscript. In the revised version we have included the functional assay to determine transepidermal water loss (TEWL). TEWL is inversely related to skin barrier function. We assessed transepidermal water loss (TEWL) in JunB mutant skin using TEWA meter. We found that JunB mutant mice revealed enhanced epidermal water loss indicating poor barrier function as compared to good barrier function in the skin of wild type mice (**Figure S3D)**.

iv) In Figure 4, the authors indicate that the differentiation of hair follicle stem cells is impaired in JunB mutant skin. To make such a conclusion, the authors have to carry out further experiments, such as double staining of CD34 with FABP5 and LRIG1 with FABP5. Also, expression analyses of Sox9 and Keratin 15, as well as stemness and quiescence markers are needed.

Reply: This is a valid concern raised by Reviewer 2. To support our claim, we have performed double immunostaining with FABP5 (red), indicative of sebaceous glands and the bulge stem cell marker Sox9 (green). We did not observe any double positive stem cell population. These results suggest that hair follicle stem cells from JunB mutant mice do not differentiate into fat producing cells. Our conclusion made from Figure 4A (in the earlier version) was overstated and misinterpreted, possibly due to overstaining with FABP5 antibody. According to our new results, we have excluded this part from the revised manuscript.

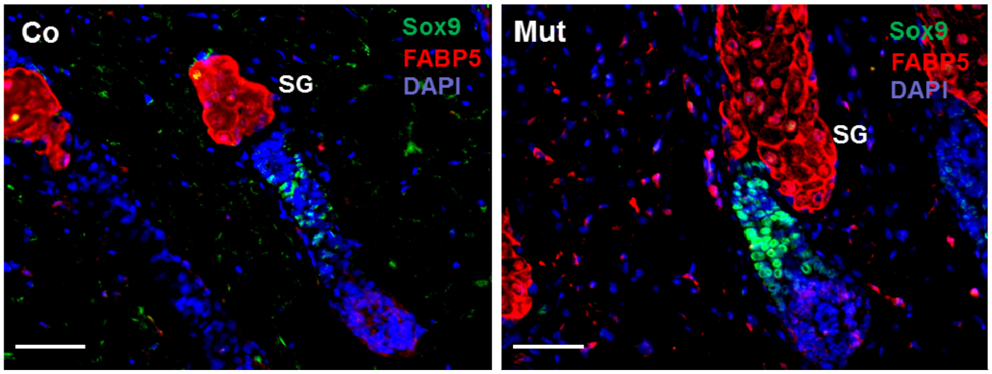

Figure 1. Representative photomicrographs with immunostaining of FABP5 (red), indicative of sebaceous glands and bulge stem cell marker Sox9 (green) in hair depilated dorsal back skin from wild type and JunB mutant mice. Nuclei stained with DAPI in blue. Scale bars, 20 µm.

v) In Figure 4B, the authors investigate the ratio of CD34 subpopulations with a6integrin and conclude that the differences observed indicate defects in maintaining stem cell homeostasis. Which part of the mouse was used for these experiments? Inflamed skin or tail skin? To make such a conclusion, *in vitro* clonogenic assays are needed. The small differences shown in the dot plots are not sufficient to make such a claim.

Reply: We appreciate Reviewer´s 2 comment. The requested information has been provided in the figure legend of **Figure 2D,** "FACS analyses from second telogen phase displaying an increased ratio of P2 to P1 of hair follicle stem cell ($CD34^{Hi}α6Itg^{Hi}/CD34^{Hi}α6Itg^{Low}$) in unperturbed back skin from 60 days old JunB mutant compared to wild type mice".

As suggested, to solidify our claim, we have performed clonogenic assays. Interestingly, FACS purified HFSCs ($CD34^{+ve}$ alpha6-$integrin^{Hi}$) from the skin of JunB mutant mice formed significantly less colonies as opposed to control HFSCs **(Figure 2C and S4C)**. These data suggest impaired self-renewal and differentiation in JunB mutant HFSCs.

Re 2d: In Figure 1 and 2 more details should be provided, such as age of mice at analysis, age when disease is complete, area of skin analyzed etc.

Reply: The details have been provided in the revised manuscript. In most experiments 60 days old mice have been used for the analyses.

Re 2d: In Figure 4A, FABP5 expression is observed not only in the sebaceous gland but also in the outer layer of the epidermis. The authors should comment on that finding. To demonstrate that FABP5 is expressed in the bulge, co-staining with CD34 or Keratin 15 is necessary.

Reply: This Reviewer is right. The answer has been described in the reply of Reviewer´s 2 comment **Re2c (iv)**.

Re 2e: The authors show that the promoters of Notch family genes have AP-1 binding sites. AP-1 family functions are dependent on dimer composition etc. and are very complex. To determine if AP-1 and in this case specifically JunB actually transcriptionally regulates Notch, Chromatin IP experiments are needed.

Reply: This is a highly appreciated, very constructive comment raised by both Reviewers. We have addressed this in our revised manuscript (as referred to in **Re1g**).

Re 2f: The authors show changes in many different pathways in JunB mutant skin through both RNA-seq

and target gene analysis, such as lipids, cholesterol, Notch pathway etc. To determine whether any of these alterations are functionally relevant, rescue experiments should be performed.

Reply: This is an essentially important advice to improve the mechanistic aspect of our manuscript. This point was also suggested by Reviewer 1. Strong activation of Notch signaling both at mRNA and protein level in JunB mutant skin especially under stress conditions was observed. Therefore, we assessed whether pharmacologic Notch blockade can restore epidermal homeostasis. Intriguingly, Notch inhibition in JunB mutant skin employing a pharmacological approach not only suppressed the lineage drift, but also restored epidermal homeostasis and skin barrier function in JunB mutant mice (**Figure 7B-7G**, revised manuscript). These rescue experiment has been included in **Figure 7** and in the result section.

Re 2g: It is recommended to improve the compensation in the dot plots in Figure 4B.

Reply: This figure **(now Figure 2D)** has been improved as suggested by Reviewer 2.

Re 2h: Markers used to identify populations such as sebaceous glands should be more consistent throughout the manuscript, e.g. FABP5 in Figure 4 and SCD1 in Figure 6.

Reply: This inconsistency was due to the availability of appropriate antibody against sebaceous glands. In case of single staining, we used the polyclonal SCD1 antibody that was generated in rabbits, while in case of double immunostaining when other primary antibody are also raised from rabbits we have to use FABP5, which was generated in goat.

Re 2i: In the discussion the authors comment that the sebaceous gland findings were not documented in Zenz et al., 2005. This is a completely different mouse model, and the JunB deletion in Keratin-5 expressing tissues was published by Meixner et al. The discussion should be changed accordingly.

Reply: We thank Reviewer 2 for this comment. The discussion has been modified as suggested.

Reviewers' comments:

Reviewer #1 (Remarks to the Author):

The authors have addressed the queries raised by including new data as requested including functional analyses of barrier function, demonstrating Jun B binding to the Notch promoter, particularly the Notch 1 & 4 promoter sites, and activation of Notch signalling in the Jun B mutants and showing impressive rescue data using Notch inhibitor particularly in respect of the Jun B mutant phenotype. As such the manuscript is significantly improved in the revised version providing strong evidence to support the notion that Jun B normally suppresses keratinocyte proliferation and impacts on Notch signalling.

I have a few concerns:
It is not clear how many times the colony forming study was performed nor why only one well is shown per condition.
How many experimental replicates were performed to get the data shown in Fig S4C?
How many times was the phenotype rescue experiment performed?

Reviewer #2 (Remarks to the Author):

The authors have addressed in the revised version all concerns raised with additional experiments and the manuscript has largely improved. I have no hesitation to recommend the revised version for publication.

Reviewer #3 (Remarks to the Author):

In the current manuscript, Singh et al describe the phenotype that emerges from the conditional ablation of the transcription factor JunB from the Keratin-14-expressing basal epidermal progenitor cells. The authors describe a cell-fate switching towards the formation of sebaceous glands upon insults that is accompanied by a seborrheic dermatitis-like syndrome and alterations in sebaceous gland function.

The quality of the data presented in the manuscript is overall clear and underscore the role of JunB in control of epidermal stem cell differentiation and cell-fate acquisition towards the sebaceous glands lineage.

However, there are several concerns that have to be addressed by the authors before publication.

Major concerns:

In Figure 2C and S4C the authors claim impaired differentiation and self-renewal defects in JunB cKO hair follicle stem cells. But at the same time, there are no differences in HFSC number in the cKO epidermis. How do authors explain this discrepancy? To help to clarify this discrepancy the authors could investigate if there are alterations in mice hair and/or hair cycle under normal homeostatic conditions.

The authors also show the involvement of JunB in epidermal terminal differentiation (Fig. 2E, S5A-C). But there is also an expansion of the K14+ basal epidermal progenitors (Fig S5A). This is a very interesting discovery, thus the data should be accompanied by quantification of the thickening of the basal vs. suprabasal layers and basal cell proliferation (BrdU+ or Phospho-H3/Ki67 IF quantifications).

Also, the authors mention (Page 9, line 239) hyperplastic thickening of the stratum corneum. How the authors reconcile this observation with the reduction in the other suprabasal markers: Krt10 (Fig 2E) or Involucrin and Lorcrin (Fig. 5B-C)?

The authors show an impressive de novo formation of sebaceous gland upon wound healing or insult (Fg 3C-D, Fig 4). But the authors should present charts showing carful quantifications of the numbers/size of SG in the different experimental conditions. Regarding the novo formation of SG, (and, as the authors mention in the text): only lineage tracing experiments would demonstrate de novo formation. Therefore, the detection of Sox9+ cells is not enough to claim de novo formation of SG.

Description of the ATACseq protocol is missing from Material and methods: It is not clear in the figure legends neither on the Methods section which population of cells where used for the RNA-seq and ATAC-seq. Total epidermis? Sorted epidermal progenitors? Sorted SG?. This information is fundamental to understand and validate the results and should me mention in Methods sections as well as the figure legend or text.

Transcriptional profiling (Fig 6):
The authors compare the gene expression between WT and cKO in control vs. insult. But there is no analysis or data comparing WT vs. cKO in the unperturbed epidermis in the same experimental conditions. It would be very informative to analyze the consequence of the lack of JunB from the epidermis in control conditions before analyzing it in the perturbed environment. Specifically: is there an increase in pro-inflammatory genes and Notch pathway in the cKO? Or this is only evident after wounding/hair plucking?

Later, on line 382, (Fig. S8), the authors present transcriptional profiling from epidermal progenitors "- under unperturbed conditions –" (-?) isolated from dorsal skin for 60 days old mice. Is this data comparable to the RNAseq data presented in Figure 6? How the epidermal progenitors where isolated/sorted in this case? Is the age different? And again, additionally to the alterations in lipid metabolism, are there any alterations in inflammation signature genes or Notch pathway in the JunB cKO under unperturbed conditions?

Accessible chromatin profiling:
The authors show in figure 6F an increase in chromatin accessibility around TSSs in the JunBcKO. But this analysis is not cross-referenced with the RNAseq data that the authors produced. The authors should evaluate the accessibility of the chromatin in subgroups of genes (up-regulated/down-regulated) in the different experimental conditions from WT and cKO. This should allow the authors to dissect the specific contribution of JunB to the differences in gene expression that probably are subdivided into different groups of DEG. And, if the hypothesis of the authors regarding the repressive role of JunB is correct, they should identify a correlation with the genes that are up-regulated and where the AP-1/JunB signature was detected by ATACseq

In the same scene, the motive analysis should be performed in subgroups of differentially expressed genes to unmask JunB direct effects vs. other AP-1 member effects in the different subgroups of DEG.

In figure 6H-I, JunB ChIP-qPCR data is presented as "Fold enrichment" vs IgG control. Would be more informative to calculate the FC vs. JunB-negative region in the vicinity of the Notch gene (eg. UTRs) or a promoter from a gene that is not differentially expressed in the cKO. Additionally to this new normalization, the authors can still present the IgG signal for each set of primes. Also, a scheme of the JunB sites on the promoters of the genes would be useful at the bottom of the ChIP-qPCR charts.

The authors link the lack of JunB at Nothc1/4 promoters with the expression of the genes. Would be important to show other histone marks in the same gene promoters associated with gene

repression or activation, and investigate if there is a
gain of active marks (or a loss of repressive ones) in the cKO. This would be important to support the model where JunB acts as a repressor of Notch 1 and 4.

Finally, there are only a few examples in the literature where a member of the Activator Protein-1 (AP-1) family of TF have a repressive role (doi: 10.1074/jbc.M010307200, 10.1083/jcb.201109045, 10.1371/journal.pone.0042152). However, the authors do not discuss this. The authors should make an effort to discuss their data in the context of the repressive roll of JunB in the epidermis and in comparison to other reported systems.

Minor comments:

Would be more appropriated to use the term "JunB conditional knockout" and "JunB cKO" instead of "Mutant" to refer to the animals where JunB was conditionally ablated by the K14-Cre expression.

Fig. 1: the terms D1, D3, D5 are confusing with respect to the letters on the figure. "Day1" should help to better understand the figure.

Page 6, line 153: the authors claim that they perform JunB staining at different stages of "murine skin development", but all the data is after birth. The authors should characterize embryonic time points to make such claim.

Page 9, line 239. The authors use the term "horny layer" to refer to the outermost layer of the epidermis. The authors may consider the use of the more common term "stratum corneum".

On Figure 2E the authors show a decreases on the early suprabasal marker K10 on the JunB cKO, is there also a decreased on late differentiation markers as Lor of Flg under unperturbed conditions in the cKO?

It is known that hair cycle affects and influence many biological processes in the epidermis. Do the authors corroborate the phase of the hair cycle where experiments were performed? And, are differences in cKO sebaceous glands during the hair cycle?

Data on figure S6C is relevant and address an important point of cell proliferation in the JunB cKO. The authors should consider including (at least part) of this data in the main figure.

On page 12, line 307 the authors state that JunB "suppress differentiation towards SG". This is a very strong statement. If this is correct, how the authors explain that not every single K14+ keratinocyte become an SG in the cKO epidermis?

Line 326: the authors state that there are "46 differently expressed lipids". A different word should be used instead of "expressed"

Line 354: authors state that there is disruption of the "Kandutsch-Russel and Bloch pathway". What is the evidence for this? Is this relevant?

On page 15, the transition in the text from describing the RNA-seq data to introduce and justify the ATAC-seq could be better presented. This reviewer recommends some rewording.

Point-to-Point Answer to the Reviewers´ Comments

Reviewer #1:

The authors have addressed the queries raised by including new data as requested including functional analyses of barrier function, demonstrating Jun B binding to the Notch promoter, particularly the Notch 1 & 4 promoter sites, and activation of Notch signalling in the JunB cKO and showing impressive rescue data using Notch inhibitor particularly in respect of the JunB cKO phenotype. As such the manuscript is significantly improved in the revised version providing strong evidence to support the notion that JunB normally suppresses keratinocyte proliferation and impacts on Notch signaling. I have a few concerns:

R1.1: It is not clear how many times the colony forming study was performed nor why only one well is shown per condition.

Reply: We appreciate the Reviewer´s concern. The colony forming study was performed two times and at each time points FACS sorted HFSCs from three control and three JunB cKO mice were cultured in duplicates. In **Figure 2C,** one representative well from each group was shown. The required experimental details of repetitive experiments have now been included in the

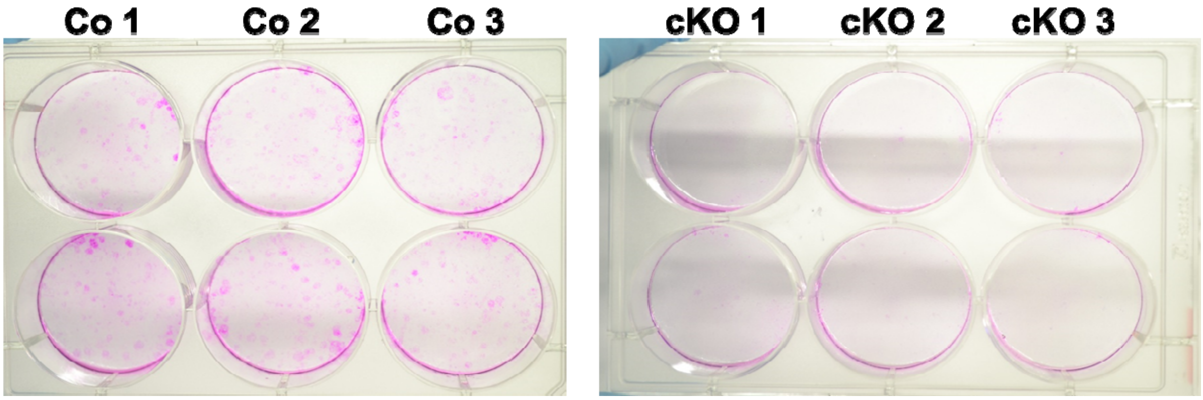

Figure 1. Shown are results of a representative colony forming unit assay out of 2 repetitive independent experiments done in duplicates from three co and three cKO HFSCs.

R1.2: How many experimental replicates were performed to get the data shown in Fig S4C?

Reply: For this data set total six experimental replicates were used.

R1.3: How many times was the phenotype rescue experiment performed?

Reply: The rescue experiments were performed two times and for each time point three mice per group were included in the experiment.

Reviewer #2:

The authors have addressed in the revised version all concerns raised with additional experiments and the manuscript has largely improved. I have no hesitation to recommend the revised version for publication.

We thank Reviewer 2 for the positive comments.

Reviewer #3:

In the current manuscript, Singh et al describe the phenotype that emerges from the conditional ablation of the transcription factor JunB from the Keratin-14-expressing basal epidermal progenitor cells. The authors describe a cell-fate switching towards the formation of sebaceous glands upon insults that is accompanied by a seborrheic dermatitis-like syndrome and alterations in sebaceous gland function.

The quality of the data presented in the manuscript is overall clear and underscore the role of JunB in control of epidermal stem cell differentiation and cell-fate acquisition towards the sebaceous glands lineage. However, there are several concerns that have to be addressed by the authors before publication.

Major concerns:

R3.1: In Figure 2C and S4C the authors claim impaired differentiation and self-renewal defects in JunB cKO hair follicle stem cells. But at the same time, there are no differences in HFSC number in the cKO epidermis. How do authors explain this discrepancy? To help to clarify this discrepancy the authors could investigate if there are alterations in mice hair and/or hair cycle under normal homeostatic conditions.

Reply:

We thank Reviewer 3 for raising this point. This discrepancy may partly be due to differences in the niche microenvironment which is distinct *in vitro* from *in vivo* conditions. In fact, employing the CFU assay *in vitro,* we observed that cKO HFSCs adopt a senescence like phenotype with large and flat morphology. This senescent phenotype is most likely due to high oxygen tension (21%) and consequently high ROS production under *in vitro* culture conditions. Even though numbers on HFSCs isolated from JunB cKO and wild type mice are unchanged, their functionality, in terms of almost absent self-renewal in the CFU assay *in vitro* was impaired. Importantly, also *in vivo* an impairment of the functionally of HFSCs in JunB cKO mice was

observed with a clearly extended resting (telogen) phase when compared to wildtype mice. This resulted in a distinctly different time line for hair cycle stages. In addition, we found a substantial impairment in the morphology of cKO hairs compared to wildtype hairs (Figure 5A).

R3.2: The authors also show the involvement of JunB in epidermal terminal differentiation (Fig. 2E, S5A-C). But there is also an expansion of the K14+ basal epidermal progenitors (Fig S5A). This is a very interesting discovery, thus the data should be accompanied by quantification of the thickening of the basal vs. suprabasal layers and basal cell proliferation (BrdU+ or Phospho-H3/Ki67 IF quantifications).

Reply: As suggested by this Reviewer, we have now included the quantification of the thickening of the basal vs. suprabasal layers in the manuscript (Supplementary Figure S5A). The quantification of basal cell (IFE) proliferation employing Ki-67 immunostaining is depicted in Supplementary Figure S6B. Our results suggest that impaired differentiation of the suprabasal layer together with enhanced basal cell proliferation in cKO account for epidermal hyperplasia.

R3.3: Also, the authors mention (Page 9, line 239) hyperplastic thickening of the stratum corneum. How the authors reconcile this observation with the reduction in the other suprabasal markers: Krt10 (Fig 2E) or Involucrin and Lorcrin (Fig. 5B-C)?

Reply: This has now been corrected from “hyperplastic thickening of the stratum corneum” to “hyperplastic thickening of the epidermis” in the revised manuscript. As we observed hyperplastic thickening of the epidermis in the cKO mice. When carefully reinvestigating histology we, in fact, found a decreased thickness of the stratum corneum. We are grateful to the Reviewer that she/he has mentioned this discrepancy.

R3.4: The authors show an impressive *de novo* formation of sebaceous gland upon wound healing or insult (Fig 3C-D, Fig 4). But the authors should present charts showing careful quantifications of the numbers/size of SG in the different experimental conditions. Regarding the novo formation of SG, (and, as the authors mention in the text): only lineage tracing experiments would demonstrate de novo formation. Therefore, the detection of Sox9+ cells is not enough to claim de novo formation of SG.

Reply: We thank Reviewer 3 for this highly constructive suggestion. We have now included the quantifications of the size (Supplementary Figure S4E and S4F) and numbers (Supplementary Figure S7A) of SG in the revised version of the manuscript. We agree with Reviewer 3 that, the detection of Sox9+ cells is not enough to claim de novo formation of SG.

R3.5: Description of the ATACseq protocol is missing from Material and methods: It is not clear in the figure legends neither on the Methods section which population of cells where used for the RNA-seq and ATAC-seq. Total epidermis? Sorted epidermal progenitors? Sorted SG? This information is fundamental to understand and validate the results and should be mention in Methods sections as well as the figure legend or text.

Reply: According to the Reviewer´s suggestions, the requested experimental details have been included in the method section as well as in the figure legend. Skin samples of completely intact (untouched) and injured skin were employed for RNA-seq analyses as depicted in Figure 6A, 6B, 6D and Supplementary Figure S8A-F. In case of ATAC-seq (Supplementary Figure S6F, 6G, S9B, S10A and S10B) and ChIP assay (Figure 6H, 6I, Supplementary Figure S11A and S11B) epidermal progenitor cells following enzymatic digestion of hair plucked skin were directly subjected to analyses.

R3.6: Transcriptional profiling (Fig 6):

The authors compare the gene expression between WT and cKO in control vs. insult. But there is no analysis or data comparing WT vs. cKO in the unperturbed epidermis in the same experimental conditions. It would be very informative to analyze the consequence of the lack of JunB from the epidermis in control conditions before analyzing it in the perturbed environment. Specifically: is there an increase in pro-inflammatory genes and Notch pathway in the cKO? Or this is only evident after wounding/hair plucking?

Reply: According to this Reviewer´s suggestion**,** new heatmaps were added which show analyses of unperturbed epidermis of WT vs JunB cKO (Supplementary Figure S8E and S8F). Our analyses suggest that apart from up-regulation of Notch4 and a slight increase in Notch1, no significant changes in the expression of other Notch family genes were observed in the unperturbed epidermis of JunB cKO mice (Supplementary Figure S8E). Of note, deficiency of JunB induced the up-regulation of several inflammatory mediators even under unperturbed condition (Supplementary Figure S8F).

Changes in Notch signaling were much more pronounced in JunB cKO skin after wounding/hair plucking (Figure 6D).

R3.7: Later, on line 382, (Fig. S8), the authors present transcriptional profiling from epidermal progenitors "- under unperturbed conditions –"(-?) isolated from dorsal skin for 60 days old mice. Is this data comparable to the RNAseq data presented in Figure 6? How the epidermal progenitors where isolated/sorted in this case? Is the age different? And again, additionally to

the alterations in lipid metabolism, are there any alterations in inflammation signature genes or Notch pathway in the JunBcKO under unperturbed conditions?

Reply: The transcriptional profiling under both conditions was performed in the dorsal skin from wild type and JunB cKO mice. The age of mice was matched in case of unperturbed and hair plucking experiments. In case of wound healing experiments, mice were 30 days older as we inflicted wounds in 60 days old mice and collected wounds at the age of 90 days. Please also see our reply to comment **R3.6.**

R3.8: Accessible chromatin profiling:

The authors show in figure 6F an increase in chromatin accessibility around TSSs in the JunBcKO. But this analysis is not cross-referenced with the RNAseq data that the authors produced. The authors should evaluate the accessibility of the chromatin in subgroups of genes (up-regulated/down-regulated) in the different experimental conditions from WT and cKO. This should allow the authors to dissect the specific contribution of JunB to the differences in gene expression that probably are subdivided into different groups of DEG. And, if the hypothesis of the authors regarding the repressive role of JunB is correct, they should identify a correlation with the genes that are up-regulated and where the AP-1/JunB signature was detected by ATACseq.

Reply: The authors would like to express their gratitude to the Reviewer for her/his constructive and highly interesting comment. In the revised manuscript, we have followed the Reviewer's suggestion. Chromatin accessibility of all peaks was presented in Figure 6F. In Supplementary Figure S10B, chromatin accessibility was shown for two different sets of genes, those which were up-regulated and those which were downregulated in JunB cKO compared with wildtype.

R3.9: In the same scene, the motive analysis should be performed in subgroups of differentially expressed genes to unmask JunB direct effects vs. other AP-1 member effects in the different subgroups of DEG.

Reply: According to the Reviewer's suggestions, ATACSeq *de novo* motifs analyses from JunB cKO have now been included in Supplementary Figure S9B by addressing two sets of differentially expressed genes. In Figure 6G, the AP1 motif was searched for in all the open chromatin conditions from both control and JunB cKO. In Supplementary Figure S9B of revised manuscript, the motifs were searched and presented separately as highly expressed and lower expressed gene sets in JunB cKO. We found that AP1 motifs are highly enriched in both highly expressed and lower expressed gene sets, suggesting an important role of JunB and other AP1 family members in the regulation of these gene sets.

R3.10: In figure 6H-I, JunB ChIP-qPCR data is presented as “Fold enrichment” vs IgG control. Would be more informative to calculate the FC vs. JunB-negative region in the vicinity of the Notch gene (eg. UTRs) or a promoter from a gene that is not differentially expressed in the cKO. Additionally to this new normalization, the authors can still present the IgG signal for each set of primes. Also, a scheme of the JunB sites on the promoters of the genes would be useful at the bottom of the ChIP-qPCR charts.

Reply: This is, indeed, a very good suggestion. We calculated the fold change by comparing the Ct values of each primer sets (JunB/AP1 binding site) with a JunB/AP1 negative region (3` UTR in case of Notch1 and 5´ UTR in case of Notch4) (Figure 6H and 6I).

R3.11: The authors link the lack of JunB at Notch1/4 promoters with the expression of the genes. Would be important to show other histone marks in the same gene promoters associated with gene repression or activation, and investigate if there is a gain of active marks (or a loss of repressive ones) in the cKO. This would be important to support the model where JunB acts as a repressor of Notch 1 and 4.

Reply: We thank Reviewer 3 for her/his constructive suggestion. We have performed ChIP-qPCR assay with other histone marks to dissect whether JunB acts as a repressor of Notch1 and Notch4. We found a reduction in repressive histone marks (H3K27Me3 and H3K9Me3) and a distinct gain of active histone marks (H3K9Ac and H3K4Me) within the promoter region of the Notch1 and Notch4 genes in basal epidermal progenitor cells of JunB cKO mice when compared to wildtype basal epidermal progenitor cells (Supplementary Figure S11A and S11B). These data imply a repressive role of JunB on Notch transcription.

R3.12: Finally, there are only a few examples in the literature where a member of the Activator Protein-1 (AP-1) family of TF have a repressive role (doi: 10.1074/jbc.M010307200, 10.1083/jcb.201109045, 10.1371/journal.pone.0042152). However, the authors do not discuss this. The authors should make an effort to discuss their data in the context of the repressive role of JunB in the epidermis and in comparison to other reported systems.

Reply: We have followed the Reviewer´s suggestion and now discussed our data implying a repressive role of JunB/AP1 family in comparison to other reported systems. Page 20……. Our results identified a specific enrichment of the JunB/AP1 motif during epidermal differentiation, which directly suppress Notch signaling via physical interaction with the Notch1 and Notch4 promoter. AP-1 has also been implicated in transcriptional repression of matrix metalloproteinase-9 through the recruitment of histone deacetylase-1 in response to interferon β

[48] and 17a-hydroxylase-17,20-lyase via blocking transcriptional activity of the nuclear receptor steroidogenic factor-1 [49]. In addition, anti-proliferative actions of members of the JunB/AP1 family have been reported through direct activation of cycle check point p16 expression [50].

Minor comments:

R3.13: Would be more appropriated to use the term "JunB conditional knockout" and "JunB cKO" instead of "Mutant" to refer to the animals where JunB was conditionally ablated by the K14-Cre expression.

Reply: This is a reasonable suggestion and we have now referred to "JunB conditional knockout" and "JunB cKO" instead of "Mutant" in the revised manuscript.

R3.14: Fig. 1: the terms D1, D3, D5 are confusing with respect to the letters on the figure. "Day1" should help to better understand the figure.

Reply: This has now been changed as suggested.

R3.15: Page 6, line 153: the authors claim that they perform JunB staining at different stages of "murine skin development", but all the data is after birth. The authors should characterize embryonic time points to make such claim.

Reply: The Reviewer is right. We now replaced the term "murine skin development" with "postnatal murine skin of young mice".

R3.16: Page 9, line 239. The authors use the term "horny layer" to refer to the outermost layer of the epidermis. The authors may consider the use of the more common term "stratum corneum".

Reply: The text has been changed as suggested.

R3.17: On Figure 2E the authors show a decreases on the early suprabasal marker K10 on the JunB cKO, is there also a decreased on late differentiation markers as Lor of Flg under unperturbed conditions in the cKO?

Reply: Yes, we also observed reduced expression of late differentiation markers in unperturbed JunB cKO skin compared to wildtype skin. However, the difference of reduced differentiation marker expression was much more pronounced in the presence of growth stimulating factors.

R3.18: It is known that hair cycle affects and influence many biological processes in the epidermis. Do the authors corroborate the phase of the hair cycle where experiments were performed? And, are differences in cKO sebaceous glands during the hair cycle?

Reply: Yes, these analyses have been performed during the telogen phase of hair cycle and in all cases the comparison were made between age-matched wild type and JunB cKO mice.

R3.19: Data on figure S6C is relevant and address an important point of cell proliferation in the JunB cKO. The authors should consider including (at least part) of this data in the main figure.
Reply: We are grateful for the suggestion, we have now moved parts of Supplementary Figure S6C to main Figure 3C.

R3.20: On page 12, line 307 the author's state that JunB "suppress differentiation towards SG". This is a very strong statement. If this is correct, how the authors explain that not every single K14+ keratinocyte become an SG in the cKO epidermis?
Reply: This was a bit overstated and now has been corrected in the revised version of our manuscript.

R3.21: Line 326: the authors state that there are "46 differently expressed lipids". A different word should be used instead of "expressed"
Reply: This term has been changed to "46 differently bio-synthesized lipids" in the revised manuscript.

R3.22: Line 354: authors state that there is disruption of the "Kandutsch-Russel and Bloch pathway". What is the evidence for this? Is this relevant?
Reply: This has been concluded from cholesterol abundance in the cKO as described in Figure 5E. As cholesterol biosynthesis in cells takes place by the Kandutsch-Russell and the Bloch pathway and lipid analyses from cKO hairs revealed marked alterations in the abundance in cholesterol derivatives, we suggest that this is at least indirect evidence for a disruption of the "Kandutsch-Russel and Bloch pathway".

R3.23: On page 15, the transition in the text from describing the RNA-seq data to introduce and justify the ATAC-seq could be better presented. This reviewer recommends some rewording.
Reply: A very constructive suggestion, we have now modified the manuscript accordingly. Page 15……… Next, to discover the potential role for JunB/AP1 in the regulation of differentially expressed genes and accessibility to gene-regulatory chromatin regions, an assay for transposase accessible chromatin with high-throughput sequencing (ATAC-seq) analysis was employed.

REVIEWERS' COMMENTS:

Reviewer #1 (Remarks to the Author):

The authors have clarified all queries raised by this reviewer improving the quality of the manuscript substantially.

Reviewer #3 (Remarks to the Author):

In the revised version of the manuscript, the authors provide new quantifications and analysis as requested. These clarify several points as the size and number of the sebaceous gland in the JunB cKO and the genes signatures in the cKO. The authors also addressed important points regarding the analysis and the comparison of the chromatin accessibility (ATAC-seq). In this reviewer opinion, only 3 minor points should be addressed:

1. The authors successfully generated new quantifications of the size of SG, but this data is only presented in supplementary figures (S4E, S4F, S7A). I think the authors should consider including the number of the quantifications in the main figures as is important and high-quality data.

2. The differences in chromatin accessibility measured by ATAC-seq in the Up-regulated vs. down-regulated genes is impressive (Fig S10B); is a pity this data is not presented in the main figure.

3. Figure 5H and 5I: The cartoon of the Notch1 and 4 promoters should be much better presented and make an effort to show the proportional distance of the primers to the TSS of the gene (that should be clearly marked with an arrow). This reviewer suggests taking a look at Figure 2C in 17344414 (DOI: 10.1101/gad.415507).

Point-to-Point Answer to the Reviewers´ Comments

Reviewer #3 (Remarks to the Author):

In the revised version of the manuscript, the authors provide new quantifications and analysis as requested. These clarify several points as the size and number of the sebaceous gland in the JunB cKO and the genes signatures in the cKO. The authors also addressed important points regarding the analysis and the comparison of the chromatin accessibility (ATAC-seq). In this reviewer opinion, only 3 minor points should be addressed:

R3.1. The authors successfully generated new quantifications of the size of SG, but this data is only presented in supplementary figures (S4E, S4F, and S7A). I think the authors should consider including the number of the quantifications in the main figures as is important and high-quality data.

Reply: As suggested by the Reviewer 3, we have now moved quantifications of size and number of SG to the respective figures (Fig 4C, 4D and Fig 7A).

R3.2. The differences in chromatin accessibility measured by ATAC-seq in the Up-regulated vs. down-regulated genes is impressive (Fig S10B); is a pity this data is not presented in the main figure.

Reply: We also share the Reviewer's concern, but due to space limitation in Fig 6 we have to move part of ATAC-seq results to the Supplementary Fig S10B.

R3.3. Figure 5H and 5I: The cartoon of the Notch1 and 4 promoters should be much better presented and make an effort to show the proportional distance of the primers to the TSS of the gene (that should be clearly marked with an arrow). This reviewer suggests taking a look at Figure 2C in 17344414 (DOI: 10.1101/gad.415507).

Reply: We thank Reviewer 3 for this constructive suggestion, we have now modified cartoon accordingly in Figure 5H and 5I.
